# Overcoming Biophysical Barriers in Melanoma Photomedicine: From Photodynamic Therapy to Smart Nanodelivery and Photoimmunotherapy

**DOI:** 10.3390/ijms27188156

**Published:** 2026-09-13

**Authors:** Francesco Russano, Luigi Dall’Olmo, Davide Brugnolo, Francesco Callegarin, Marcodomenico Mazza, Paolo Del Fiore, Rocco Caminiti, Marco Rastrelli, Simone Mocellin

**Affiliations:** 1 Soft-Tissue, Peritoneum and Melanoma Surgical Oncology Unit, Veneto Institute of Oncology IOV—IRCCS, 35128 Padua, Italy; francesco.russano@iov.veneto.it (F.R.); luigi.dallolmo@unipd.it (L.D.); marcodomenico.mazza@iov.veneto.it (M.M.); paolo.delfiore@iov.veneto.it (P.D.F.); marco.rastrelli@unipd.it (M.R.); simone.mocellin@unipd.it (S.M.); 2 Department of Surgery, Oncology and Gastroenterology (DISCOG), University of Padua, 35128 Padua, Italy; davide.brugnolo@iov.veneto.it; 3 Clinical Research Unit, Veneto Institute of Oncology IOV—IRCCS, 35128 Padua, Italy; 4 Casa di Cura Caminiti, Villa San Giovanni, 89018 Reggio Calabria, Italy; rocco.caminiti@icloud.it

**Keywords:** photodynamic therapy, melanoma, nanotechnology, photoimmunotherapy, microneedles, tumor microenvironment, targeted drug delivery, immunogenic cell death

## Abstract

Malignant melanoma presents formidable therapeutic challenges due to optical shielding and free-radical scavenging by endogenous melanin, profound tumor microenvironment hypoxia, and aggressive metastatic dissemination, rendering conventional photodynamic therapy (PDT) clinically immature. This narrative review comprehensively synthesizes literature across PubMed/MEDLINE, Scopus, and Web of Science evaluating photochemical mechanisms, photosensitizing agents, bioengineered drug delivery systems, and adjacent light-triggered strategies. Preclinical evidence demonstrates that third-generation photosensitizers, targeted organic/inorganic nanoparticles, and transdermal dissolving microneedle (MN) arrays effectively bypass the stratum corneum, enhance drug bioavailability, and alleviate hypoxia-mediated treatment resistance. Furthermore, femtosecond two-photon PDT converts melanin into an active energy-transfer mediator, while nanotechnology-driven photoimmunotherapy (PIT) triggers immunogenic cell death (ICD) and systemic CD8+ cytotoxic T-lymphocyte activation to induce abscopal regression of un-irradiated distant metastases. Nevertheless, critical translational bottlenecks persist, including an overwhelming reliance on static two-dimensional (2D) cell cultures, a lack of validated prognostic or predictive biomarkers, and a complete absence of randomized controlled clinical trials. Ultimately, advancing melanoma photomedicine from bench to bedside requires standardized photophysical dosimetry, systematic evaluation in multicellular three-dimensional (3D) tumor spheroids, and prospective clinical trials defining its role in multimodal dermato-oncology.

## 1. Introduction

Melanoma occupies a distinct position within dermatologic oncology because its biology differs substantially from the keratinocyte-derived lesions for which topical Photodynamic Therapy (PDT) is most established. Across the reviewed literature, PDT is consistently defined as a photochemical treatment requiring a photosensitizer (PS), an appropriate light source, and molecular oxygen, with cytotoxicity mediated mainly through reactive oxygen species (ROS) and singlet oxygen generation [1,2]. This mechanism is attractive for cutaneous disease because the skin is externally accessible to light and can be treated with spatial precision; however, malignant melanoma is not simply another superficial skin tumor. Unlike keratinocyte-derived non-melanoma skin cancers (NMSCs), such as basal cell carcinoma (BCC) and cutaneous squamous cell carcinoma (cSCC), where topical photodynamic therapy (PDT) exhibits well-established clearance rates, melanoma represents the most aggressive cutaneous malignancy, accounting for over 80% of skin cancer mortalities due to its rapid vertical dermal invasion and high metastatic propensity [3,4,5]. Melanin pigmentation, marked intratumoral heterogeneity, deep tissue infiltration, tumor microenvironment (TME) hypoxia, and complex immune escape mechanisms collectively modify and complicate the therapeutic problem [3,4,5,6]. Optically and biochemically, melanin acts as a major physical barrier; it absorbs light across a broad ultraviolet-visible spectrum (peaking around 335 nm and attenuating light transmission beyond 700 nm), directly competing with PS for photon absorption within the therapeutic window [3,5,6]. Furthermore, melanin functions as an endogenous antioxidant, free-radical stabilizer, and ROS scavenger, while melanosomes within melanoma cells provide an intracellular protective shield that sequesters therapeutics and drives multidrug resistance [4,6]. Consequently, low-pigmented or amelanotic melanoma cells demonstrate significantly greater susceptibility to PDT-induced apoptosis than heavily pigmented melanotic lines [5,6]. Microenvironmental barriers further impede treatment success: solid melanoma lesions develop severe hypoxia and dense extracellular matrix (ECM) networks, which severely deplete the molecular oxygen required for cytotoxic singlet oxygen (^1^O_2_) generation via oxygen-dependent Type II photochemical reactions while inducing adaptive antioxidant defense pathways [4,5,6]. Finally, restricted photon penetration depth in deeply infiltrating nodular lesions, combined with systemic metastatic dissemination, limits localized photodynamic destruction and permits tumor evasion from host immune surveillance [3,5,6].

The historical and general rationale for PDT in skin cancer was addressed in bibliometric, mechanistic, and dermatology reviews. Sun et al. described the growth of PDT research in skin cancer and summarized the central antitumor mechanisms as cytotoxicity, microvascular injury, and immune activation [1]. Balakirski et al. described PDT in dermatology as a modality built around protoporphyrin IX (PpIX) formation after 5-aminolevulinic acid (5-ALA) or methyl aminolevulinate administration, followed by light-induced ROS generation [2]. Broader oncologic and dermato-oncologic overviews similarly positioned PDT as an evolving, highly adaptable anticancer modality spanning fundamental mechanistic research and clinical translation across a wide spectrum of malignancies [7,8,9]. At the basic research level, modern PDT (“PDT 2.0”) leverages sophisticated photochemical mechanisms, wherein light-activated PS interact with molecular oxygen to generate cytotoxic ROS, inducing direct tumor cell death via apoptosis and necrosis, localized vascular shutdown, and robust immunogenic responses within the TME [7,8]. Beyond basic photochemical principles, recent dermato-oncologic and broader oncologic syntheses emphasize the rapid emergence of third-generation PS and nanotheranostic delivery vehicles (e.g., liposomes, polymeric micelles, silica, upconversion nanoparticles, and antibody-conjugated smart carriers), which optimize water solubility, tumor selectivity, and deep-tissue light absorption while enabling real-time diagnostic monitoring [8,9]. Clinically, PDT has achieved established regulatory approvals and ongoing phase I–III clinical evaluation for superficial keratinocyte-derived cutaneous lesions, such as actinic keratoses and Bowen’s disease, as well as solid organ malignancies, including non-small cell lung cancer, head and neck carcinomas, prostate carcinoma, and breast cancer recurrences [7,8,9]. Furthermore, recent translational paradigms highlight the clinical integration of daylight-PDT protocols to minimize procedural pain, the use of advanced light technologies (such as diode lasers and fractional laser pre-treatments), and synergistic combinations with chemotherapy, targeted therapies, or immune checkpoint blockade (photoimmunotherapy), underscoring PDT’s transition from a niche localized intervention to a versatile component of modern multimodal oncology [7,8,9]. For melanoma specifically, Naidoo et al. and Nkune and Abrahamse framed PDT as a potential alternative or adjunctive strategy for metastatic melanoma, while also emphasizing the need for improved PS and delivery systems [3,6].

To address these distinct mechanisms rigorously, this review establishes a clear taxonomy separating conventional, oxygen-dependent PDT, which relies strictly on photosensitizer-mediated photochemical reactive oxygen species (ROS) and singlet oxygen (^1^O_2_) generation, from adjacent photo-mediated strategies, including photothermal therapy (PTT), chemo-photothermal platforms, photoimmunotherapy (PIT), and photoacoustic cellular lysis. While all these modalities share the requirement for optical activation, they operate through fundamentally divergent physical, photophysical, and biological pathways. Conflating photochemical and photothermal or photomechanical processes masks critical differences in therapeutic efficacy, microenvironmental sensitivity (e.g., hypoxia dependence), and translational bottlenecks. Consequently, each modality is categorized and evaluated independently across all sections according to its specific photophysical driver and therapeutic mechanism.

## 2. Methods

A comprehensive literature search was conducted across PubMed/MEDLINE, Scopus, Web of Science, and Google Scholar to identify peer-reviewed publications. The search strategy utilized targeted combinations of Medical Subject Headings (MeSH) and free-text keywords joined by Boolean operators (AND/OR). Core search parameter strings encompassed combinations of: (“Photodynamic Therapy” OR “PDT” OR “Photosensitizer”) AND (“Melanoma” OR “Cutaneous Melanoma” OR “Melanotic Melanoma”) AND (“Nanotechnology” OR “Nanoparticles” OR “Microneedles” OR “Transdermal” OR “Photoimmunotherapy” OR “Photothermal” OR “Photoacoustic”). Eligible publications were evaluated based on pre-established thematic criteria designed to capture foundational photochemical principles, bioengineered drug delivery systems, and adjacent photo-mediated modalities. Inclusion criteria encompassed: (1) original research articles, clinical trials, and comprehensive reviews focused on photodynamic or light-triggered interventions in malignant melanoma; (2) preclinical investigations spanning 2D cell monolayers, 3D multicellular tumor spheroids, and in vivo animal models; and (3) mechanistic studies detailing Type I/Type II photochemical pathways, ROS quantum yields, nanocarrier pharmacokinetics, transdermal microneedle arrays, or immunogenic cell death (ICD) cascades. Conversely, exclusion criteria comprised: (1) studies focusing exclusively on non-melanoma skin cancers (such as basal cell carcinoma or cutaneous squamous cell carcinoma) without direct applicability to melanoma biology; (2) investigations lacking explicit light dosimetry parameters, wavelength characterization, or photosensitizer identification; and (3) conference abstracts, editorial letters, non-peer-reviewed preprints, and non-accessible full texts. In accordance with narrative review methodology, study selection was guided by thematic relevance to the biophysical barriers and bioengineered solutions of melanoma photomedicine rather than an exhaustive PRISMA-guided systematic screening. Following this structured criteria-based evaluation, a total of 79 peer-reviewed publications were selected, critically analyzed, and synthesized within this review.

## 3. Mechanistic Basis of PDT and Its Relevance to Melanoma

**Figure 1:**
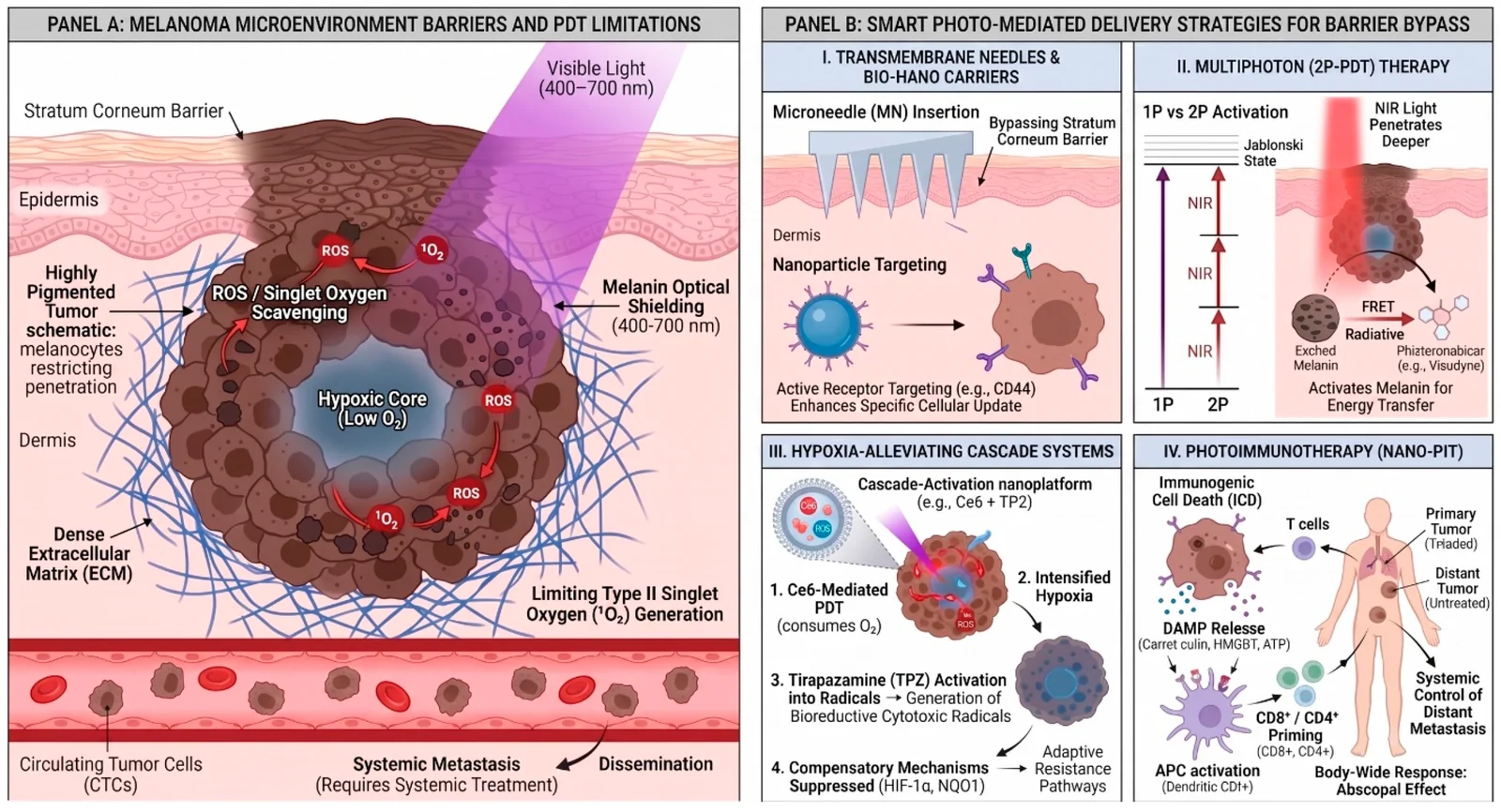
Schematic overview of biophysical barriers limiting photodynamic therapy (PDT) in malignant melanoma and bioengineered strategies for barrier bypass. (**Panel A**) Melanoma Microenvironment Barriers and PDT Limitations: The stratum corneum acts as a primary physical barrier to topical drug delivery. Within the tumor microenvironment, endogenous melanin pigment exerts optical shielding by absorbing light across the visible spectrum (400–700 nm) and functions as a reactive oxygen species (ROS) scavenger. Dense extracellular matrix (ECM) and severe core hypoxia limit oxygen-dependent Type II singlet oxygen (O 12) generation, while hematogenous dissemination of circulating tumor cells (CTCs) promotes systemic metastasis. (**Panel B**) Smart Photo-Mediated Delivery Strategies for Barrier Bypass: (**I**) Transdermal microneedle (MN) arrays physically bypass the stratum corneum, while receptor-targeted nanocarriers (e.g., anti-CD44) enhance cell-specific endocytosis. (**II**) Multiphoton two-photon (2P-PDT) activation utilizes near-infrared (NIR) light for deeper tissue penetration, converting excited melanin into an energy transfer mediator via Förster resonance energy transfer (FRET) and radiative mechanisms. (**III**) Hypoxia-alleviating cascade platforms (e.g., Ce6 + TPZ) exploit light-induced oxygen consumption to intensify local hypoxia, selectively activating bioreductive prodrugs (tirapazamine) and suppressing adaptive survival pathways (HIF-1α, NQO1). (**IV**) Nanotechnology-driven photoimmunotherapy (Nano-PIT) triggers immunogenic cell death (ICD) and damage-associated molecular pattern (DAMP) release, driving dendritic cell activation and CD8+/CD4+ T-cell priming to induce systemic antitumor immunity and abscopal regression of distant metastases. (Created with biorender.com).

The core PDT mechanism in the reviewed literature begins with PS excitation by light. After absorption of photons, the PS reaches an excited state and can participate in type I or type II photochemical reactions. Type I reactions involve electron or hydrogen transfer and formation of free radicals, whereas type II reactions generate singlet oxygen through energy transfer to molecular oxygen [2,6]. The generated ROS and singlet oxygen (^1^O_2_) inflict severe oxidative damage on essential cellular biomolecules, including structural proteins, membrane lipids, and nucleic acids, as well as subcellular organelles such as the mitochondria, endoplasmic reticulum (ER), lysosomes, and plasma membrane [6]. Depending on the PS’s subcellular localization, physicochemical properties, tissue bioavailability, molecular oxygen concentration, and light parameters (fluence and wavelength), this localized photodamage activates distinct or concurrent cell death pathways, including regulated apoptosis, necrosis, autophagy, or mixed death phenotypes [6]. Specifically, photodamage directed at the mitochondria predominantly triggers caspase-mediated apoptotic cascades, whereas severe disruption of plasma membrane integrity or drastic intracellular ATP depletion shifts the response toward necrotic cell death or autophagic degradation [6]. Melanoma creates distinct optical and biological constraints for PDT. Nkune and Abrahamse highlight that melanin acts as a primary mediator of PDT resistance by serving as a physical optical shield that absorbs light within the critical 500–600 nm wavelength range, thereby impairing photon delivery to target tissues. Biochemically, melanin functions as a potent antioxidant and ROS scavenger, directly neutralizing PDT-generated cytotoxic radicals. Comparative preclinical studies demonstrate that melanoma cell lines with lower melanin expression (such as A375 amelanotic cells) exhibit significantly higher susceptibility to PDT-induced cell death compared to heavily pigmented melanotic lines (such as B16F1 and B16F10). Consequently, strategies capable of inhibiting melanogenesis or inducing cellular depigmentation are recognized as essential interventions to enhance the therapeutic efficacy of PDT in malignant melanoma [6]. This makes pigmentation not merely a diagnostic feature, but a potential determinant of photodynamic response. Pires et al. [10] approached the problem differently by developing a multiphoton PDT (2p-PDT) strategy utilizing ultrafast (~100 fs) near-infrared (NIR) laser light. By comparing clonally identical melanotic (B16F10) and amelanotic (B78H1) melanoma lines, they demonstrated that while conventional single-photon (1p-PDT) responses are equivalent regardless of pigmentation, 2p activation unexpectedly exhibits dramatically heightened cytotoxicity in pigmented cells. Specifically, using the clinically approved PS Visudyne, which possesses a low two-photon absorption cross-section (σ2p ~ 31 GM), 2p-PDT achieved a greater than 6-fold reduction in LD50 in melanotic compared to amelanotic cells. Mechanistically, ultrafast NIR pulses induce multiphoton (2p and 3p) absorption by melanin, generating broadband fluorescence emission that overlaps by ~67% with Visudyne’s absorption spectrum [10]. This enables efficient energy transfer (via radiative reabsorption and/or Förster resonance energy transfer, FRET) to the PS, accompanied by photomechanical melanosomal disruption and the localized release of cytotoxic melanin precursors and ROS. Translational feasibility was established in an in vivo murine model of conjunctival melanoma, where raster-scanned 2p-PDT with Visudyne (0.8 mg/Kg) achieved complete tumor eradication with minimal collateral ocular damage. Consequently, this approach shifts the longstanding therapeutic paradigm, converting melanin from a light-attenuating obstacle into an active energy-transfer mediator for targeted photodynamic destruction [10]. Oxygen availability is another mechanistic issue [4,6]. PDT requires molecular oxygen, and Nkune and Abrahamse describe the hypoxic TME as a major contributor to PDT failure in metastatic melanoma, where severe oxygen scarcity limits Type II photodynamic cytotoxicity and promotes tumor growth, invasion, and metastasis [6]. Compounding microenvironmental hypoxia, continuous photodynamic action rapidly depletes local molecular oxygen (3O2). Modern bioengineering approaches address this limitation by coupling PS with hypoxia-activated bioreductive prodrugs (such as tirapazamine), effectively turning treatment-induced oxygen depletion into a secondary cytotoxic trigger [11]. Although this approach remains preclinical, it directly reflects a recurring melanoma problem: the photodynamic effect depends on oxygen, whereas solid TME may be oxygen-poor, demonstrating how bioengineered combination platforms can turn hypoxia into a therapeutic advantage [6,11]. Immune responses represent another critical dimension of photodynamic intervention. Dudzik et al. [12] and Falk-Mahapatra and Gollnick [13] delineate PDT as a potent trigger of local acute inflammation and systemic antitumor immunity. Beyond direct cytotoxicity, local photodynamic stress serves as a key driver of acute inflammation and host immune recognition. By triggering ICD and releasing damage-associated molecular patterns, PDT can potentially convert localized photodamage into systemic antitumor immunity, as detailed in dedicated photoimmunotherapy paradigms [12,13]. For melanoma, a classically immunogenic malignancy, these immunological mechanisms are conceptually crucial, particularly because several melanoma-directed phototherapeutic strategies combine local light activation with exogenously administered immunostimulatory agonists or photothermal platforms [4,14,15]. Examples in the literature range from in situ photoimmunotherapy combining indocyanine green (ICG) and laser irradiation with topical imiquimod [15] to nanoplatform-based photothermal immunotherapy incorporating TLR-7 agonists [14] or thermal hydrogels [16]. However, despite compelling mechanistic evidence and preliminary clinical feasibility studies [15], the reviewed literature does not supply randomized controlled clinical trials proving that PDT-induced antitumor immunity yields statistically significant overall survival or durable progression-free survival benefits in melanoma patients (Figure 1) [12,13,15].

## 4. Melanoma Biology, Prognostic Context and Implications for PDT Development

Ding et al. comprehensively reviewed the prognostic biomarker landscape in cutaneous melanoma, emphasizing that classical clinicopathological variables integrated into the 8th edition AJCC staging system, such as Breslow thickness, ulceration, mitotic rate, and regional lymph node status, fail to provide sufficient individual accuracy for predicting metastatic risk, as evidenced by the ~2% 10-year mortality observed even among thin, early-stage IA melanomas [17]. To refine risk stratification, emerging non-histological biomarkers have been explored across multiple biological compartments [17]. Serum lactate dehydrogenase (LDH) remains the sole serologic biomarker formally incorporated into AJCC stage IV staging, functioning as a surrogate of metabolic cell turnover and disease burden under hypoxic microenvironmental stress [17]. However, other serological markers show significant clinical potential: S100B, a calcium-binding protein regulating cell cycle progression, serves as a sensitive indicator of relapse incorporated into European clinical guidelines, while melanoma inhibitory activity (MIA), a 12-kDa secreted protein promoting metastasis via ECM detachment, demonstrates superior diagnostic sensitivity compared to conventional liver enzymes [17]. Beyond serology, circulating tumor products (CTPs) provide real-time liquid biopsy metrics, encompassing circulating tumor cells (CTCs), cell-free DNA (cfDNA), circulating tumor DNA (ctDNA harboring BRAF/NRAS mutations), and circulating exosomal microRNAs (e.g., exo-miR-532-5p and exo-miR-106b) [17]. Furthermore, commercially available gene expression profiling (GEP) panels (such as DecisionDx-Melanoma and the Merlin assay) have emerged to stratify recurrence and nodal metastatic risk [17]. Complementing liquid and bulk tissue assays, Martinek et al. developed a spatial transcriptomics pipeline using multiplex immunofluorescence-guided laser capture microdissection (IF-guided LCM) [18]. By utilizing an Arcturus XT system equipped with a non-destructive infrared laser and RNA-preserving IF staining (RIN > 8), this methodology allows the precise harvesting of phenotypically defined single cells, including gp100/Mart1+ melanoma cells, CD3+ T lymphocytes, and CD14+ monocytes/macrophages, directly from intact tumor nests versus stromal compartments without disrupting local tissue microarchitecture [18]. Although this extensive biomarker literature does not directly define approved photodynamic indications, its relevance to melanoma photomedicine is profound: light-based therapies operate within a disease framework heavily governed by metastatic dissemination, microenvironmental spatial topology, cellular heterogeneity, and immune evasion [17,18]. Connecting dissemination biology with optical interventions, label-free photoacoustic technologies have been developed to target pigmented CTCs in real time during hematogenous transit, exploiting melanosome absorption to achieve selective photomechanical cell lysis and reduce distant metastatic seeding [19]. Despite these significant diagnostic and technological advancements, the reviewed literature reveals a critical translational gap: no validated predictive biomarkers or patient-selection algorithms currently exist to guide melanoma phototherapy [17,18,19]. Neither the serologic, exosomal, and spatial transcriptomic signatures identified by Ding et al. and Martinek et al. [17,18], nor the optical flow cytography metrics demonstrated by He et al. [19], have been established as clinical criteria to stratify which patients should receive conventional PDT, nanoparticle-enhanced PDT, femtosecond pulsed 2p-PDT, photothermal therapy, or photoimmunotherapy. Bridging this gap will require prospective clinical trials integrating spatial biomarker profiling and CTC monitoring to identify patient subsets most likely to benefit from targeted light-based interventions [17,18,19].

## 5. Photosensitizers and Conventional PDT in Melanoma

PDT relies on light-induced excitation of a PS to generate cytotoxic ROS, primarily singlet oxygen (^1^O_2_), within a therapeutic window of 600–800 nm. In malignant melanoma, target accumulation and photon penetration are severely constrained by melanin’s optical attenuation and radical scavenging capacity, alongside melanosomal drug sequestration. To overcome these constraints, PS development has progressed through distinct chemical generations and nanostructured delivery systems [20,21]. First-generation PS, such as hematoporphyrin derivative (HpD) and Photofrin, suffer from major clinical drawbacks, including poor chemical purity, weak absorption at wavelengths above 600 nm, slow systemic clearance, and prolonged, highly debilitating skin photosensitivity [6,20]. Second-generation PS, including porphyrins, chlorins, and phthalocyanines, were developed to optimize these properties, exhibiting stronger absorption in the red and NIR regions (600–700 nm) to maximize tissue penetration [6,20]. Phthalocyanines (Pcs), in particular, are highly valued for their high singlet oxygen quantum yields and long triplet-state lifetimes, especially when coordinated with central diamagnetic metal ions such as zinc, aluminum, or magnesium, which promote intersystem crossing [6,20]. However, their clinical utility is hindered by their high lipophilicity, which leads to self-aggregation in aqueous physiological media and drastically reduces their fluorescence and singlet oxygen production [6,20]. To address this, third-generation PS encapsulate second-generation agents within advanced organic or inorganic nanocarriers or conjugate them to tumor-specific targeting ligands (such as antibodies or peptides) to improve solubility, prevent aggregation, and enhance selective accumulation in melanoma tissues [6]. In recent years, researchers have also explored alternative, non-porphyrin or naturally derived PS to diversify the chemical space and exploit novel light delivery mechanisms [20]. A prime example is Cherenkov Radiation induced PDT (CR-PDT), an innovative strategy designed to bypass the physical limits of light penetration through tissue and melanin [22]. This approach repurposes older-generation dyes like Eosin, which absorbs at 524 nm in the green spectrum, a region normally considered unsuitable for deep-tissue external illumination [22]. By pairing Eosin or its synthesized Eosin-DOTAGA conjugate with positron-emitting radionuclides ^18^F-FDG, ^68^Ga, ^90^Y, the internal blue-weighted Cherenkov luminescence emitted during radioactive decay acts as an in situ light source [22]. This luminescence excites the Eosin molecule via Cherenkov Radiation Energy Transfer (CRET), generating cytotoxic singlet oxygen in complete darkness at deep tissue sites, eliminating the need for external laser light [22]. Similarly, Riboflavin (Vitamin B2) is a biocompatible, natural blue-light-absorbing pigment, but its therapeutic utility is severely limited by rapid photodegradation [21]. To circumvent this stability bottleneck, researchers developed the acetylated derivative 3-methyltetraacetyl-riboflavin (3MeTARF), which exhibits an outstanding photostability with a photolysis quantum yield at least two orders of magnitude lower than that of free riboflavin [21]. Upon blue-light irradiation (450 nm), 3MeTARF generates robust intracellular oxidative stress that activates MAPK signaling through the phosphorylation of p38 and JNK, triggering Caspase-3/7 activation, PARP cleavage, and apoptotic cell death in WM115 and A375 melanoma cells [21]. Furthermore, the natural pigment Hypericin, derived from *Hypericum perforatum*, boasts an exceptionally high singlet oxygen quantum yield but is plagued by poor water solubility and systemic phototoxicity [21,23]. Advanced nanomedicine has resolved this by using *Hypericum perforatum*-derived exosome-like nanovesicles (HPDENs), natural nanocarriers that encapsulate hypericin at a ratio of 1.03% [23]. Upon yellow LED light activation (598 nm), HPDENs generate ROS through dual Type I (superoxide, hydroxyl radicals) and Type II (singlet oxygen) pathways, resulting in high cellular uptake, a strong concentration-dependent reduction in WM-266-4 cell viability (IC_50_ = 6.669 µg/mL), and extensive apoptotic cell death characterized by the upregulation of BAX and cleavage of Caspase-3 [23]. In vivo studies further demonstrate that HPDEN-mediated PDT significantly suppresses tumor growth and induces extensive tumor necrosis without inducing any systemic organ or biochemically detectable liver and kidney toxicity, offering a highly biocompatible and effective theranostic platform [23]. The integration of nanotechnology has further revolutionized PDT delivery by providing a diverse array of organic and inorganic nanocarriers designed to optimize PS delivery [6,20]. Organic systems such as liposomes, micelles, and polymeric nanoparticles, most notably poly(lactic-co-glycolic acid) (PLGA) nanoparticles, improve the solubility of hydrophobic agents and promote their accumulation in tumors through the enhanced permeability and retention (EPR) effect [3,6,20]. For example, the encapsulation of PpIX or zinc phthalocyanine tetrasulfonate in PLGA nanoparticles preserves their singlet oxygen quantum yield while dramatically enhancing cellular uptake and tumor cytotoxicity in B16F10 melanoma models [6,20]. To bypass the iron-dependent biotransformation of PpIX into HEME, which acts as a major biological feedback loop, membrane fusion liposomes (MFLs) co-encapsulating 5-ALA and the iron chelator deferoxamine have been designed [20]. These liposomes enter the cytoplasm directly through membrane fusion, releasing their cargo to simultaneously boost PpIX accumulation and block DNA repair, resulting in significant tumor mass reduction in female C57 mice [20]. Natural, cost-effective plant-virus-based scaffolds have also been developed; for instance, tobacco mosaic virus (TMV) nanorods have been used to encapsulate the cationic porphyrin Zn-EpPor, stabilizing its structure and achieving a 40% increase in mitochondrial uptake [6,20]. Lipid-based nanocarriers, including solid lipid nanoparticles and surface-modified liposomes, have also been investigated to enhance transdermal skin penetration and overcome the systemic clearance of hydrophobic PS [20]. Similarly, negatively charged ICG molecules have been stabilized by encapsulation in liposomes coated with the positively charged polysaccharide chitosan, increasing transdermal delivery and skin permeation while generating potent phototoxic activity under 775 nm laser irradiation [20]. Nanotechnology also enables highly synergistic, multimodal platforms, such as theranostic nanoscale metal–organic frameworks (NMOFs) like PCN-222, which co-encapsulates iodine-substituted BODIPY to prevent self-quenching and achieve a remarkable 10,000-fold increase in light-to-dark cytotoxicity [20]. Active targeting is achieved by conjugating these nanoparticles to monoclonal antibodies (mAbs), such as anti-DR5 antibodies, or to ligands like hyaluronic acid (HA), which selectively bind to CD44 receptors overexpressed on malignant melanocytes, thereby minimizing off-target toxicity [6,20]. To bridge this translational gap, future research must clearly distinguish conventional oxygen-dependent PDT from adjacent photo-mediated strategies, such as photothermal therapy, photoimmunotherapy, and chemo-photothermal platforms, which rely on local heat or immune checkpoint inhibitors rather than pure PS-oxygen photochemistry. Overcoming the biophysical limitations of melanoma will ultimately require standardized multicenter clinical trials, the optimization of combination regimens that address hypoxia, and the deployment of advanced delivery devices such as transdermal MN, daylight-simulating LED arrays, or light-emitting fabric devices to ensure uniform, pain-free therapeutic illumination [24]. The methodological constraints, preclinical 3D modeling requirements, and translational gaps associated with these platforms are comprehensively discussed in Section 10.

## 6. Nanoparticle-Mediated PDT for Melanoma

The clinical management of malignant melanoma represents one of the most formidable challenges in contemporary dermato-oncology [6,25]. Characterized by a high metastatic propensity, genetic heterogeneity, and an innate resistance to standard therapeutic regimens such as dacarbazine-based chemotherapy and conventional ionizing radiation, melanoma accounts for over 80% of all skin cancer-related mortalities despite representing a small fraction of cutaneous malignancies [6,26,27,28]. PDT has garnered clinical interest as a non-invasive, spatially controllable alternative that exploits the cytotoxic reaction of a photoexcited PS in the presence of molecular oxygen to generate singlet oxygen (^1^O_2_) and other ROS [6,25,26,29]. However, conventional PDT efficacy in melanoma is severely hampered by major biological and biophysical barriers, including the optical shielding and antioxidant scavenging of endogenous melanin, systemic clearance, poor water solubility of classical PSs, and the severely hypoxic and acidic microenvironment of solid melanoma lesions [6,26,29]. To overcome these historical limitations, the integration of nanotechnology has emerged as a revolutionary paradigm, driving the transition from second-generation PS to advanced, targeted third-generation drug delivery platforms [6,26,30,31]. Nanoparticles (NPs) offer unique biophysical properties, such as high surface-area-to-volume ratios, ease of surface modification, protection of the payload against premature systemic degradation, and tunable drug-release kinetics, that maximize intra-tumoral PS deposition while dramatically minimizing off-target toxicities to surrounding healthy tissues (Table 1) [6,26,31].

**Table 1:** Overview of advanced nanocarriers, targeted systems, and transdermal MN platforms evaluated for phototherapy in melanoma.

Delivery Category	Platform/Nanostructure	Primary Modality & Driver	Biological/Microenvironmental Target	Experimental Validation Model	Key Outcomes & Therapeutic Efficacy
Organic Nanocarriers	SLN-AlPc/ClAlPc-SLNs	PDT (Photochemical O 12)	Passive tumor accumulation (EPR effect)	In vitro (B16F10 melanoma cell monolayer)	3.2-fold higher phototoxicity compared to free ClAlPc.
Inorganic/Carbon Dots	Fe-CDs@Ce6	PDT + Ferroptosis (Dual ROS/Fenton)	Intracellular GSH depletion/TME redox state	In vivo (Subcutaneous B16 murine melanoma)	GSH depletion, Fenton reaction amplification, near-complete tumor ablation, and pulmonary metastasis suppression.
Active Targeted Systems	Ac-HA-PFC-Ppa	Oxygenating PDT (Photochemical O 12)	Surface CD44 receptors/Intratumoral core hypoxia	In vivo (OM431 choroidal melanoma xenograft)	Ameliorates core hypoxia via PFC oxygen delivery, reducing mean tumor weight to 0.05 g.
Active Targeted Systems	scFv–SNAP-tag Bioconjugates	Targeted PDT (Photochemical O 12)	CSPG4 cell-surface proteoglycan	In vitro (Human/murine melanoma cell lines)	Enables high stoichiometric ligand binding, preventing off-target systemic phototoxicity.
Stimuli-Responsive Systems	IR780@PCPNs	Combined PDT/PTT (Photochemical + Heat)	TME extracellular acidity (pH 6.5–5.0)	In vitro/In vivo (B16F10 murine melanoma)	Acidity-triggered PEG detachment, 54.6% photothermal conversion efficiency, on-demand IR780 release.
Biomimetic/Multi-Death	M-Cu@Fh	Photo-PIT/Ion Death (Immunogenic)	MHC-I membrane/Intracellular GSH/FDX1 balance	In vivo (B16F10 murine melanoma model)	Synchronized ferroptosis/cuproptosis, MHC-I upregulation, robust CD8+ T-cell infiltration.
Dissolving Microneedles (MNs)	TF-TA-PPa MNs	Chemo-PDT (Photochemical + Prodrug)	Stratum corneum bypass/ROS-cleavable linker	Ex vivo (Rat skin)/In vivo (Murine model)	Deep penetration (600 µm depth), 89.2% tumor inhibition via light-triggered Tegafur release.
Hypoxia-Defying MNs	MN-ZnBP	Photoredox Catalysis (Oxygen-independent)	Cellular NADH redox balance	In vivo (Hypoxic murine melanoma model)	Oxygen-independent photoredox catalysis, potent antiangiogenesis, and complete tumor ablation.

### 6.1. Passive Targeting and the EPR Effect

Passive targeted nanoparticle delivery exploits the pathophysiological features of melanoma tissues, particularly the presence of hyperpermeable, leaky tumor blood vessels and highly compromised lymphatic drainage systems [6,26]. This phenomenon, known as the EPR effect, allows nanoscale carriers (typically in the size range of 10 to 200 nm) to selectively extravasate from the bloodstream and accumulate within the interstitial spaces of the tumor mass [6,26].

#### 6.1.1. Organic Nanocarriers

Solid Lipid Nanoparticles (SLNs): Solid lipid nanoparticles are highly biocompatible, solid-lipid-based colloidal carriers that excel at carrying hydrophobic compounds in their oily cores [6,30]. Preclinical work has demonstrated the therapeutic potential of Amazon butter-based solid lipid nanoparticles loaded with aluminum phthalocyanine (SLN-AlPc) [30]. This biomimetic formulation showed high colloidal stability, prolonged drug release, and excellent phototoxicity against B16-F10 murine melanoma cells in vitro by promoting targeted subcellular damage and cellular immune activation [26,30]. Similarly, aluminum chloride phthalocyanine (ClAlPc)-loaded solid lipid nanoparticles (ClAlPc/SLNs) exhibited a 3.2-fold higher phototoxicity against pigmented B16-F10 melanoma cells compared to free ClAlPc [6,26].

Liposomes and Chitosan-Coated Nanoformulations: Liposomes offer robust, biocompatible shells capable of encapsulating both hydrophilic and lipophilic agents [6,31]. Chitosan-coated liposomes have been engineered to address the inherent instability, self-aggregation, and negative charge of ICG [6,26,31]. The positive surface charge of the chitosan coating induces strong electrostatic interactions with the cell membranes, significantly promoting cellular uptake, enhancing skin permeation, and stabilizing ICG, which translates to a two-fold increase in ICG bioavailability and robust photodynamic destruction under NIR laser irradiation [6,26,31].

Polymeric Micelles: Polymeric micelles, consisting of amphiphilic diblock copolymers like PEG-b-PLGA, self-assemble into a core–shell structure that stabilizes water-insoluble PSs in aqueous physiological fluids [6,26,31]. A notable co-delivery platform based on mPEG-b-PLGA micelles was developed to simultaneously encapsulate the PS IR-768 and the chemotherapeutic agent daunorubicin [6,26,31]. This dual-drug-loaded polymeric micellar system acted synergistically, dramatically increasing the intracellular generation of singlet oxygen during PDT in A375 human melanoma cells while overcoming chemoresistance mechanisms [6,26,31]. Similarly, PpIX-loaded polymersomes have been combined with cold atmospheric plasma (CAP) as an alternative light activation source, inducing small pore formation in A375 cell membranes to facilitate nanoparticles cellular internalization, resulting in the selective destruction of 80% of malignant cells without harming normal dermal fibroblasts [26].

#### 6.1.2. Inorganic and Carbon-Based Nanoparticles

Inorganic nanoparticles provide precise physical structures, high loading capacities, and distinct diagnostic/therapeutic functionalities [6].

Carbon Dots and Carbon Quantum Dots (CQDs): CQDs are a highly photostable, water-soluble class of nanomaterials possessing strong, tunable photoluminescence, making them excellent candidates for concurrent PDT and bioimaging (theranostics) [32]. In a study by Mehravanfar et al., hydrothermal-synthesized N-doped CQD nanoparticles were used to load Indocyanine Green (icG@cQD) via physisorption, increasing particle stability and resistance to photobleaching [32]. The resulting icG@cQD formulation achieved a quantum yield of approximately 53%, enhanced photostability by up to 35% over 14 days, and significantly reduced B16F10 melanoma cell survival in vitro to 28% compared to 48% for free ICG upon 808 nm laser irradiation [32]. In vivo, the icG@cQD nanoparticles accumulated extensively in the tumor margins of C57BL/6 mice, delivering a potent, localized antitumor effect with negligible photothermal or systemic toxicity [32].

Fe Ions-Doped Carbon Dots (Fe-CDs): To combine photodynamic action with other regulated cell death pathways, Li et al. developed chlorin e6-modified Fe ions-doped carbon dots (Fe-CDs@Ce6) [33]. This platform exploits the elevated glutathione (GSH) levels in melanoma cells to reduce Fe3+ into redox-active Fe2+, which subsequently catalyzes the Fenton reaction to generate hydroxyl radicals (OH•), thus triggering ferroptosis [33]. When combined with 660 nm laser irradiation, the Fe-CDs@Ce6 nanoplatform exhibited a synergistic therapeutic effect where the PDT-induced GSH depletion directly amplified the Fenton-driven lipid peroxidation and ferroptotic signaling pathways, resulting in the near-complete ablation of subcutaneous B16 tumors in vivo and the inhibition of pulmonary metastasis without causing any systemic toxicity [33].

Chitosan-Stabilized Dietary PS: High biocompatibility has also been achieved using natural, dietary chlorophyll derivatives [25]. Shinde et al. synthesized sodium copper chlorophyllin-loaded chitosan nanoparticles (CH-SCC NPs) via ionic gelation [25]. This formulation stabilized the natural pigment, improved its bioavailability, and achieved an 80–85% cell kill rate in B16 melanoma cells under a portable, low-cost green laser pointer (532 nm), demonstrating outstanding biocompatibility in zebrafish embryos and chick chorioallantoic membrane (CAM) assays [25].

### 6.2. Active Targeting: Ligands, Receptors, and Recombinant Fusion Proteins

While passive delivery via the EPR effect significantly improves intra-tumoral accumulation, it lacks the cellular selectivity required to exclusively discriminate between malignant melanocytes and adjacent healthy cells [6]. To address this, active targeting strategies modify the surface of nanocarriers with target-specific ligands, such as mAbs, antibody fragments, aptamers, peptides, folic acid, or carbohydrates, that bind with high affinity to receptors selectively overexpressed on the plasma membranes of melanoma cells [6,26,27].

#### 6.2.1. Key Melanoma Antigens and Receptors

Cutaneous metastatic melanoma cells express a distinct set of surface markers that have been exploited as active targeting handles [6]. These include:

Chondroitin Sulfate Proteoglycan 4 (CSPG4): Highly overexpressed in melanoma cells, CSPG4 is a transmembrane proteoglycan that supports tumor cell migration, invasion, and metastatic progression, making it a primary target for antibody-directed delivery [27].

MIA Antigen: Expressed by malignant melanocytes to promote detachment and invasion, MIA acts as a reliable target for functionalized nanoparticles, such as gold nanoparticles conjugated with anti-MIA antibodies and zinc phthalocyanine [6,27].

CD44 Receptors: The CD44 receptor is a transmembrane glycoprotein overexpressed on melanoma stem-like cells that binds avidly to HA [6,26].

Integrins (especially αβ_3_): Recognized by the cyclic Arginine-Glycine-Aspartic (cRGD) peptide motif [6,26].

#### 6.2.2. Active Preclinical Targeted Formulations

HA-Based Systems: Active CD44 targeting was implemented by Li et al. by grafting fluorinated perfluorocarbons (PFCs) and the PS pyropheophorbide a (Ppa) onto an acetylated HA backbone, forming self-assembling amphiphilic micelles (Ac-HA-PFC-Ppa) [6,26]. The HA shell actively recognized CD44 receptors on choroidal melanoma cells (OM431), drastically enhancing cellular endocytosis, while the internal fluorinated segments dissolved and transported oxygen directly to the hypoxic core of the tumor [6,26,27]. This dual-action micellar platform ameliorated microenvironmental hypoxia and reduced tumor weight to 0.05 g in vivo [6,26].

Peptide-Conjugated Systems: Active peptide-directed targeting was exemplified by Sebak et al., who conjugated a cyclic cRGD peptide to the surface of PLGA-lipid hybrid nanoparticles encapsulating the PS tri-sodium ferrous chlorophyllin (Fe-CHL) [26]. The cRGD ligand facilitated receptor-mediated endocytosis, directing the Fe-CHL cargo to B16-F10 cells and inducing robust singlet oxygen generation upon laser irradiation at 652 nm [26].

Recombinant Fragment and SNAP-Tag Technology: To resolve the structural and economic limitations of whole mAbs, Malindi et al. proposed the use of recombinant antibody fragments (such as single-chain variable fragments, scFvs) genetically fused to a SNAP-tag platform [27]. The SNAP-tag is a modified DNA-repair enzyme (O^6^-alkylguanine-DNA alkyltransferase) that reacts covalently with benzylguanine-modified substrates, providing a highly versatile and chemically precise protein-labeling platform [27]. This allows scFvs targeting melanoma markers like CSPG4 to be linked with high stoichiometry to PS or silver nanoparticles (AgNPs) [27]. The resulting bioconjugates optimize biodistribution, promote active uptake, and enable targeted photo-induced damage without passive leakage or off-target systemic phototoxicity [27].

Lectin-Mediated Glycan Targeting: Choi et al. engineered hollow gold-iron oxide nanoparticles conjugated with Sambucus nigra lectin (SNA) to target sialic acid (glycans) overexpressed on B16F10 melanoma membranes [34]. Upon NIR laser activation, the SNA-functionalized nanoparticles not only mediated localized hyperthermia but also effectively blocked the immune-evasive CD24 “don’t eat me” signal, successfully promoting the phagocytosis and elimination of metastatic melanoma spheroids by surrounding M2 macrophages [34].

### 6.3. Microenvironmental Responsiveness: Stimuli-Triggered and Synergistic Hybrid Nanoplatforms

The melanoma TME is characterized by severe hypoxia, dense ECM, and a hallmark extracellular acidity (pH 6.5–5.0) resulting from upregulated glycolytic metabolism (the Warburg effect) [28,29]. Advanced nanomedicine has harnessed these physiological abnormalities to design stimuli-responsive and synergistic hybrid nanoplatforms that activate or disassemble exclusively in response to local microenvironmental triggers [26,28].

#### 6.3.1. Acidity-Triggered PEG Detachment (The PEG Dilemma Resolved)

Although PEGylation of nanoparticles is necessary to prolong bloodstream circulation and bypass macrophage clearance, the dense, hydrophilic PEG corona physically blocks PS release and impairs endocytic uptake once the nanocarrier reaches the tumor site [28]. To conquer this “PEG dilemma,” Tsai et al. designed acidity-triggered PEG-detachable hybrid nanoparticles consisting of a polydopamine (PDA) core and a PEGylated chitosan shell linked via an acid-labile benzoic imine bond (IR780@PCPNs) [28]. At physiological pH (7.4), the nanoparticles remained stable and shielded [28]. However, upon entering the acidic melanoma microenvironment (pH 6.5–5.0), the benzoic imine bond rapidly hydrolyzed, shedding the hydrophilic PEG layer and exposing a positively charged chitosan surface [28]. This charge reversal vastly accelerated cellular internalization by B16F10 cells [28]. Furthermore, the acidic pH increased the protonation of the amine groups of chitosan and phenolic hydroxyls of PDA, disrupting the hydrophobic and pi-pi interactions binding the PS IR780, resulting in rapid, on-demand drug release [28]. Under 808 nm NIR laser irradiation, the internalized IR780@PCPNs achieved a photothermal conversion efficiency of 54.6% and generated abundant singlet oxygen, successfully obliterating mitochondrial integrity and inducing apoptosis [28].

#### 6.3.2. Hypoxia-Alleviating and Chemo-PDT Co-Delivery

Amorphous Calcium Carbonate Platforms: To exploit pH responsiveness while co-delivering chemotherapeutics and photodynamic agents, Yakubova et al. fabricated biodegradable amorphous calcium carbonate nanoparticles (CaCO_3_ NPs) co-loaded with doxorubicin (Dox) and chlorin e6 (Ce6) [35]. When administered intratumorally or systemically, the CaCO_3_ shell rapidly dissolved in the acidic TME, releasing the Dox and Ce6 payloads [35]. Under 650 nm laser irradiation, the localized release of Ce6 generated high ROS concentrations, which acted synergistically with doxorubicin-induced DNA damage to achieve a 94% tumor volume reduction, highlighting the superior efficacy of localized intratumoral delivery compared to systemic administration [35].

#### 6.3.3. Multi-Regulated Cell Death: Ferroptosis, Cuproptosis, and Photoinduced Charge Transfer

Blue-Light Activated Cu@Ferrihydrite (M-Cu@Fh): An innovative, biomimetic platform was designed by Rong Yang et al. by encapsulating copper-doped ferrihydrite nanoparticles inside a melanoma-derived MHC-I-overexpressing cell membrane (M-Cu@Fh) [36]. Activated by biocompatible blue light (450 nm), the M-Cu@Fh nanoparticles triggered a synchronized intracellular release of Fe2+ and Cu2+ [36]. This ion surge induced a novel dual-regulated cell death pathway: the Fe2+ depleted GSH and downregulated GPX4 to drive ferroptosis, while the Cu2+ induced abnormal oligomerization of dihydrolipoamide S-acetyltransferase (DLAT) and downregulated FDX1 to trigger cuproptosis [36]. Simultaneously, the blue-light irradiation activated the NFkB pathway to upregulate MHC-I expression in B16-F10 tumors, recruiting and activating cytotoxic CD8+ T lymphocytes and DCs to establish systemic, long-term anti-tumor immunity [36].

Organic Heterojunction Nanoparticles (PP NPs): To completely circumvent the oxygen-dependent triplet-state limitations of traditional Type II PS, Jin et al. developed organic heterojunction nanoparticles composed of the conjugated polymer donor PFBT and the electron acceptor PCBM (PP NPs) [29]. Under light irradiation, the PP NPs exhibited an ultra-efficient photoinduced charge transfer (97.7%), generating long-lived radical species, PCB Mullet^−^, that initiated direct electron and hole transfer reactions [29]. This Type I-dominated process produced massive quantities of superoxide and hydroxyl radicals while alleviating the strict oxygen requirement of conventional PDT [29]. Remarkably, rather than acting as a physical shield, endogenous melanin acted as a photocatalytic accelerator in this system, donating electrons to fill the oxidative holes of the PP heterojunction, thereby sustaining a continuous, amplified redox cycle that achieved a 90% tumor growth inhibition rate in preclinical in vivo B16F10 murine models [29].

#### 6.3.4. Nanofiber Hybrids and Exosome Release

Melanin Nanoparticle-Nanofiber Hybrids (NNHs): Kabay et al. formulated stimuli-responsive nanoparticle-nanofiber hybrids by embedding natural eumelanin nanoparticles (MNPs) into a polycaprolactone (PCL) electrospun nanofiber matrix [37]. Under UV-A irradiation, the MNPs consumed local oxygen to release superoxide and hydrogen peroxide, achieving a sustained, zero-order release of co-loaded ampicillin and inducing up to 37% melanoma cell death, establishing the feasibility of photoactive transdermal patches [37].

PDT-Induced Exosome Dynamics: The biological consequence of nanoparticle-mediated PDT also extends to the extracellular space [38]. PDT has been shown to trigger a massive release of extracellular vesicles and melanoma cell-derived exosomes (MTEX) into the TME [38]. These post-PDT exosomes carry distinct immunogenic cargo and damage-associated molecular patterns (DAMPs) that can stimulate dendritic cell maturation and reverse the epithelial-to-mesenchymal transition (EMT), altering the immunological status of the surrounding TME [38].

## 7. MN and Transdermal Delivery Strategies

### 7.1. Biological Barriers of the Skin and the MN Paradigm

The cutaneous environment presents a highly formidable biophysical and biochemical barrier that severely restricts the topical penetration and localized accumulation of therapeutic agents. The outermost layer of the skin, the stratum corneum, is a highly organized lipophilic matrix of keratin-rich corneocytes embedded within a lamellar lipid bilayer, which acts as a rate-limiting barrier preventing the passive transport of molecules larger than 500 Da or those with unfavorable hydrophilic properties [39,40,41,42]. Malignant melanoma originates from the neoplastic transformation of melanocytes situated in the deeper, basal layer of the epidermis [43]. The aggressive, deeply penetrating, and highly metastatic nature of melanoma requires localized, deep, and uniform therapeutic exposure to ensure the complete eradication of both primary tumor masses and infiltrating micro-lesions [41,44,45]. Traditional clinical interventions, such as surgical resection, are often disabling or inappropriate for mucosal, acral, or cosmetically sensitive lesions, whereas conventional chemotherapy and systemic immunotherapy are severely limited by off-target toxicities, rapid systemic clearance, and poor accumulation at the tumor site [39,41,43,44,45,46,47,48].

To bypass these limitations, MN arrays have emerged as a revolutionary transdermal drug delivery system. Typically consisting of microscale projections ranging from 150 to 1500 μm in height, MNs possess the unique mechanical capability to pierce the stratum corneum, forming hundreds of transient microscopic channels or pathways [40,41,42,44,45,46,49]. These micropores facilitate the passive and controlled diffusion of diverse cargo, including chemotherapeutics, PS, nucleic acids, and macromolecular vaccines, directly into the viable epidermis and dermis, bypassing the hepatic first-pass metabolism and gastrointestinal degradation [40,41,42,44,45,46]. Crucially, because MNs are designed to avoid reaching the deeper dermal nociceptors and blood vessels, their application is completely non-invasive, safe, and virtually painless, which significantly enhances patient compliance compared to traditional subcutaneous or intratumoral injections [40,41,42,44,45,46]. Based on their structure and release mechanisms, MN are conventionally classified into solid, coated, hollow, dissolving, and hydrogel-forming systems [41]. Among these, dissolving and hydrogel-forming MN fabricated from biocompatible, water-soluble, or swellable polymers, such as HA, polyvinylpyrrolidone, and poly(vinyl alcohol), have attracted the greatest interest due to their high cargo capacity, excellent biosafety profiles, and ability to release payloads on demand without generating sharp biohazardous waste [40,41,42,44,45,46,49].

### 7.2. Dissolving MN Systems for Photodynamic and Synergistic Therapy

Dissolving MN represent a highly advanced transdermal platform, as they dissolve rapidly upon contact with the skin’s interstitial fluid, releasing encapsulated nanoformulations directly into the malignant tissue [40,41,44,45,46,49]. Anning Li et al. (2026) developed an innovative NIR-responsive transdermal platform consisting of soluble MN that co-deliver the chemotherapeutic tegafur and the second-generation PS pyropheophorbide a (PPa) [44]. This system utilizes a bifunctional prodrug (TF-TA-PPa) wherein tegafur and PPa are covalently linked via a ROS-cleavable thioether bond [44]. Due to the poor water solubility of the conjugate, the prodrug was encapsulated within Pluronic F68 polymeric micelles to form a stable colloidal dispersion of approximately 113 nm [44]. This micellar system was subsequently loaded into the tip layer of dissolving MN fabricated from polyvinylpyrrolidone K90, protamine sulfate, and the cell-penetrating peptide octa-arginine (Arg8) [44]. The resulting MN demonstrated exceptional mechanical resilience, exceeding 3.6 N per needle, and successfully penetrated rat skin to a depth of 600 μm in the dermis [44]. Upon 808 nm laser irradiation, the PPa moiety generates a robust intracellular and extracellular cascade of ROS [44]. This localized oxidative stress cleaves the adjacent thioether linkage on demand, accelerating the hydrolysis of the ester bond and releasing active tegafur (TF-OH) directly at the tumor site, yielding an 89.2% tumor growth inhibition rate in preclinical in vivo rodent models with negligible systemic toxicity [44]. The strategic integration of cell-penetrating peptides (CPPs) such as octa-arginine in the polymeric matrix significantly enhances cellular internalization and tissue permeation of the prodrug [44].

Similarly, Fan Liu et al. (2023) developed a photoactivatable, dissolving MN system (MN-pB/I) using a polyvinylpyrrolidone matrix containing nanoliposomes co-loaded with the PS ICG and an ROS-activatable doxorubicin prodrug (pB-DOX) [45]. Concentrated at the needle tips through vacuum-assisted casting [45], the liposomes are rapidly released as the PVP matrix dissolves, with 92.2% of the drug payload penetrating the stratum corneum into the viable epidermis and dermis within 5 min [45]. When exposed to an 808 nm laser, ICG initiates PDT by generating ROS, which cleaves the prodrug moiety to release active doxorubicin, while also exerting photothermal effects that enhance the permeability and cellular uptake of the liposomes [45]. Furthermore, because of ICG’s strong optical absorbance, this platform provides dual-modal fluorescence and photoacoustic imaging guidance to monitor treatment efficacy [45]. This highly coordinated chemo-phototherapy system achieved a 93.5% tumor growth inhibition rate in preclinical in vivo B16F10 murine models following a single application, with no detectable cardiotoxicity or systemic side effects in animal cohorts [45].

To address the severe challenges of the hypoxic TME, which typically limits the efficacy of oxygen-dependent PDT, Han Zhang et al. (2025) constructed an electrostatic force-enabled MN patch (MN-ZnBP) [46]. This design exploits the strong intermolecular electrostatic interactions between a cationic, non-pyrrolic seco-chlorin zinc PS, ZnBP(w), and a negatively charged HA matrix [46]. This electrostatic coordination drastically increased the mechanical strength of the needles (>0.15 N/needle) [46], prevented PS self-aggregation, and maximized drug-loading efficiency [46]. Once released, ZnBP(w) targets and binds to biological electron donors, such as 1,4-dihydronicotinamide adenine dinucleotide (NADH), via electrostatic and supporting interactions [46]. Under red light irradiation (640–660 nm), ZnBP(w) initiates highly efficient photoinduced electron transfer (PET) with NADH, disrupting cellular NADH/NAD+ redox homeostasis and inducing tumor cell apoptosis and necroptosis even under extreme hypoxic conditions [46]. This MN-delivered photoredox catalysis achieved potent antiangiogenesis in cockscomb models and robust tumor ablation in melanoma mouse models, while systematically reducing systemic phototoxicity and photosensitivity compared to intravenous administration [46]. Furthermore, to address microenvironmental hypoxia, Ma et al. engineered a dissolving polymeric MN array co-delivering chlorin e6 and the hypoxia-activated prodrug tirapazamine (TPZ). Upon 660 nm laser illumination, Ce6-mediated oxygen depletion intensifies local hypoxia, which in turn cascade-activates TPZ into toxic bioreductive radicals, destroying residual hypoxic melanoma cells while suppressing NQO1 and HIF-1α adaptive resistance pathways [11].

### 7.3. Advanced Microchannel and Vesicular Delivery Platforms

Yiping Guo et al. (2024) developed highly elastic transfersomes (TFS) co-loaded with 5-fluorouracil (5-FU) and Imperatorin (IMP) incorporated into a Carbopol 940 hydrogel matrix. Franz diffusion cell studies demonstrated that the TFS-gel achieved a steady-state rate of 5-FU 1.47 times greater and skin deposition 4.43 times higher than conventional creams. Upon UVA irradiation, the co-delivered TFS-gel generated abundant intracellular ROS, inducing G2/M phase cell cycle arrest and apoptotic disintegration in A375 malignant melanoma [48]. Topical PDT has also been significantly advanced through ultra-deformable invasomes. Antonio Gaballo et al. (2023) engineered egg-lecithin-based invasomes containing limonene to encapsulate 5-ALA. Limonene acts as a powerful penetration enhancer by fluidizing the stratum corneum lipid matrix. Diffusion kinetics revealed that these invasomes penetrated ex vivo pig skin to 500 μm depth within 3 h [43]. Evaluated in a 3D skin model comprising keratinocytes layered over agarose-embedded HBL melanoma spheroids, the invasomes crossed the epidermal barrier, accumulated inside spheroids, and converted 5-ALA into PpIX, achieving a twofold increase in photodynamic cell death upon 630 nm red light activation [43]. To optimize transdermal delivery, Ana Carolina Viana et al. (2026) developed Amazon butter-based solid lipid nanoparticles (SLNs) loaded with curcumin and formulated with oleic acid. These liquid crystalline nanodispersions (A1Cur) were incorporated into a dissolving MN system fabricated from PVA/PVP. This combination generated a robust system with high mechanical resistance to axial compression (32 N) and created microchannels up to 381 μm in depth [40]. Upon blue laser activation (425 nm), the MN-loaded nanodispersion generated superoxide and singlet oxygen, inducing high phototoxicity and apoptosis in epidermoid carcinoma cells [40]. Furthermore, alternative physical delivery membranes have been explored to precisely control PS flux. Meryem Çamur Demir et al. (2024) investigated the transdermal delivery of water-soluble indium and zinc metallophthalocyanines (InPc and ZnPc) across thermally crosslinked poly(vinyl alcohol) (PVA) membranes [39]. By optimizing temperature, concentration, and pH parameters, InPc and ZnPc release rates reached 85.36% and 69.78%, respectively, under a pH gradient of pH 1.2 in the donor compartment and pH 5.5 in the receptor compartment [39]. Photodynamic activation reduced the viability of human melanoma cells (SK-MEL-30) with IC50 values of 4.058 μg/mL (InPc) and 11.574 μg/mL (ZnPc) [39]. Additionally, Saichand Thakkellapati et al. (2025) designed a non-invasive, reusable photothermal transdermal patch by embedding silver nanoparticle-polymer composites between polyvinyl acetate layers [50]. Activated by an affordable infrared lamp (instead of high-cost lasers) [50], the photothermal temperature rise (up to 62 °C) synergistically induces ROS generation (photodynamic effect) and protein denaturation, eradicating cancer cells (glioma) and multidrug-resistant bacterial biofilms [50].

### 7.4. MN-Mediated Photo-Immunotherapy and Nanoamplifiers

PDT not only induces direct cytotoxic damage to malignant cells but also acts as a potent trigger for systemic anti-tumor immunity by inducing ICD. During ICD, dying cancer cells release tumor-associated antigens (TAAs) and damage-associated molecular patterns (DAMPs) that stimulate the recruitment and maturation of antigen-presenting cells [42,47,49]. This immunological response can be significantly amplified by using MNs to deliver immunoadjuvants, gene-silencing tools, and ion-coordinating nanoparticles directly to the skin’s rich antigen-presenting cell network [42,47,49]. Shaozhen Wang et al. (2025) synthesized peptide-modified cationic liposomes (Ce6/CpG@Lip-TD) co-encapsulating Ce6 and CpG. When topically applied in vivo, Ce6-mediated PDT under 660 nm laser irradiation induced robust ICD, promoted dendritic cell maturation, and stimulated T-lymphocyte infiltration to eradicate local and distant tumors via an abscopal effect [47]. To combine gene therapy and PDT, Yuchen Qi et al. (2026) developed a HA-based MN patch integrated with self-assembled DNA nanoflowers (Z/H@DFs) carrying PD-L1 antisense oligonucleotides (ASO), AS1411 aptamers, Hemin, and zinc phthalocyanine [42]. The AS1411 aptamer promotes nucleolin-targeted endocytosis, Hemin catalyzes $H_{2}O_{2}$ decomposition to alleviate hypoxia and boost singlet oxygen production, while PD-L1 ASO silences PD-L1 to reverse immunosuppression. When delivered via the DFMN patch, the system achieved a 3-fold increase in tumor-infiltrating CD8+ T cells and elevated systemic IFN-γ and TNF-α [42]. Furthermore, Zhouyu Jiang et al. (2024) developed a calcium-ion-modulated nanoamplifier (nanoamplifiers@MNs) comprising $Ca^{2+}$-PPA coordination nanoparticles integrated into a soluble PVPK90 MN matrix [49]. Under NIR light, PPA generates ROS to induce cell death, while released $Ca^{2+}$ triggers mitochondrial/ER calcium overload to induce pyroptosis. Simultaneously, the platform promotes M1 macrophage polarization and dendritic cell maturation to remodel the TME [49].

### 7.5. Translational Gaps, Safety Profiles, and Clinical Outlook

Despite the highly promising preclinical and mechanistic achievements of MN-mediated transdermal phototherapy, a significant translational gap remains to be addressed before these technologies can be integrated into standard clinical practice [41,44]. This translational delay is primarily driven by four critical limitations. First, almost all current experimental evaluations are conducted in small animal models, such as mice or rats, whose skin is characterized by a significantly thinner epidermal layer, much higher hair follicle density, and different viscoelastic properties compared to human skin [41]. Consequently, the mechanical strength, skin insertion kinetics, and drug diffusion profiles validated in rodent models may not accurately predict performance in human patients [41]. Second, from a clinical and systemic review standpoint, conventional PDT must be clearly and deliberately distinguished from adjacent photo-mediated strategies such as photothermal therapy (PTT), chemo-photothermal platforms, and photoimmunotherapy. While these related approaches share the requirement for light activation and local energy delivery, they rely on photothermal heating, local tissue coagulation, or immune checkpoint modulation rather than classical, oxygen-dependent PS-mediated photochemistry. Conflating these mechanisms under a single “photodynamic” umbrella compromises the quality of clinical data analysis. Third, several pharmacotechnic and safety concerns remain unresolved, including the risk of local skin irritation, infection, or allergic reactions from repeated microneedling [41], the long-term chemical and physical stability of macromolecular payloads (such as DNA, antibodies, or peptides) within the polymeric MN matrix during storage [41,49], and the challenges of scaling up the sterile fabrication of these complex, multi-layered device-drug combination systems [41,49]. Overcoming these scientific and engineering bottlenecks will require systematic, multi-disciplinary research partnerships bridging clinicians, academic material scientists, and the pharmaceutical industry [41,49].

## 8. PDT, Immune Activation and Photoimmunotherapy

### 8.1. Mechanistic Foundations of PDT-Induced Immunogenicity and ICD

The therapeutic efficacy of conventional PDT was historically attributed strictly to localized direct cytotoxicity and microvascular shutdown [12,13]. However, modern translational oncology increasingly recognizes that PDT is a potent trigger of local acute inflammation and systemic antitumor immunity, capable of transitioning a localized photodynamic injury into a systemic, tumor-specific immune response [12]. This immunogenic transition is initiated by PS-mediated ROS generation under light irradiation, which inflicts severe oxidative stress on subcellular organelles [12,13,51]. Depending on the PS’s specific localization, this localized photodamage activates ICD, a regulated cell death pathway that fundamentally differs from immunologically silent apoptosis [12,13,52]. The hallmark of ICD is the coordinated, spatiotemporally programmed exposure and release of DAMPs and alarmins into the TME. Upon photodynamic stress, CRT translocates to the outer leaflet of the plasma membrane, serving as a dominant “eat-me” signal that facilitates engulfment by professional APCs [12,13,52]. Concurrently, severe intracellular ATP depletion triggers active ATP secretion to recruit DCs and macrophages into the stroma [12,13,52]. This is accompanied by the passive release of high-mobility group box 1 (HMGB1), which binds to TLR4 on DCs to promote stable antigen presentation, alongside heat shock proteins (Hsp70/Hsp90) that provide additional adjuvant signaling [12,13,52]. This DAMP-mediated cascade initiates a robust acute inflammatory reaction in the tumor stroma. Endothelial contraction and localized vascular permeabilization allow the rapid influx of innate immune effectors, dominated by neutrophils and macrophages. Upregulation of cell adhesion molecules (E-selectin, ICAM-1) facilitates leukocyte rolling and extravasation, driven by a surge of inflammatory cytokines (TNF-α, IL-1β, IL-6) and complement activation (C3a, C5a). Neutrophils clear primary tumor debris and infiltrate tumor-draining lymph nodes to assist CD8+ T-cell priming, while macrophages release cytolytic factors upon TLR activation [12,13]. Primed by these innate responses, the adaptive arm of the host immune system is systematically engaged. Mature DCs migrate to regional lymph nodes, expressing MHC complexes, costimulatory molecules (CD80, CD86), and T-cell-priming cytokines to orchestrate the clonal expansion of CD8+ cytotoxic T lymphocytes (CTLs) and helper CD4+ T lymphocytes. CD8+ CTLs represent the absolute core effector component of PDT-induced systemic immunity; their selective depletion completely abolishes protective antimetastatic effects. NK cells further contribute by upregulating MHC class I-like molecules and NKG2D ligands on stressed cells [12,13].

### 8.2. Nanotechnology-Driven Photoimmunotherapy (Nano-PIT) Platforms

Despite the powerful immunogenic potential of classical PDT, its clinical efficacy in heavily pigmented melanoma is severely restricted by melanin-mediated light attenuation, rapid systemic PS washout, and the highly immunosuppressive nature of the melanoma microenvironment [12]. To bypass these biological constraints, nanotechnology-based photoimmunotherapy (Nano-PIT) has emerged as a major design paradigm, co-delivering photosensitizing agents, target-specific ligands, and immunomodulatory adjuvants within a single nanovehicle to elicit synergistic local and systemic antitumor activity [12,53]. Among the most sophisticated systems studied, biomineralized and carrier-free organic/inorganic nanostructures are designed to maximize target tissue delivery [12]. For example, biomineralized aluminum hydroxide nanoparticles synthesized with bovine serum albumin and loaded with Chlorin e6 (Al-BSA-Ce6 NPs) possess an ultra-small size of ~25.3 nm, promoting efficient tissue penetration and selective tumor accumulation via the EPR effect [53]. Upon 660 nm laser irradiation, these biomineralized platforms trigger significant tumor regression, mature local DCs, and stimulate a robust systemic immune response characterized by elevated serum levels of TNF-α, IL-6, IL-12, and IFN-γ [53]. Similarly, two-dimensional black phosphorus (BP) nanosheets PEGylated and combined with the synthetic TLR7 agonist imiquimod (BP-PEG NSs + R837) provide exceptional photothermal conversion alongside sustained adjuvant release, increasing splenocyte CD8+ T-cell frequencies and driving complete tumor ablation under 808 nm light [53]. This platform was further advanced by coating BP nanoparticles with phenylalanine-lysine-phenylalanine (FKF) tripeptide-modified antigen epitopes (FKF-OVAp@BP), establishing a robust long-term immunological memory that confers 100% protection against tumor re-challenge in vivo [53]. To combine physical tissue penetration with localized biochemical immune-checkpoint reversal, advanced transdermal MN arrays have been developed [12]. A core–shell dissolving microneedle (CSMN) array has been engineered to concentrate chitosan-derived nanoparticles encapsulating indocyanine green (ICG-NPs) on the MN tip shell, while loading the indoleamine 2,3-dioxygenase (IDO) inhibitor 1-methyltryptophan (1-MT) into the cross-linked poly(vinylpyrrolidone)/poly(vinyl alcohol) core [12,53]. This transdermal patch physically bypasses the stratum corneum, delivering the PS directly into the melanoma tissue. Following 808 nm laser activation, the localized PTT effect triggers robust melanoma cell lysis and a 3.9-fold increase in DC maturation [53]. Simultaneously, the sustained release of 1-MT blocks the compensatory upregulation of IDO that typically occurs post-photothermal ablation, reversing local immunosuppression and promoting a durable CD8+ T-cell response that eradicates primary B16 tumors and controls distant untreated lesions [53]. Additionally, self-assembled nanomicelle MN patches co-encapsulating the photothermal agent IR780 and the autophagy inhibitor chloroquine (C/I-Mil) achieve deep tumor penetration, destroying cellular membrane integrity, increasing the phagocytic index of macrophages by 3.0-fold, and reducing distant tumor volumes by 3.4-fold [12,53]. Targeted Nano-PIT has also leveraged natural biomimetic carriers to optimize biocompatibility and bypass off-target toxicities [12,54]. A highly innovative platform utilizes photosensitive hybrid exosomes generated by the freeze–thaw membrane fusion of human Vδ2-T cell-derived exosomes (γδ-T exosomes) and Ce6-loaded liposomes [55]. The functional surface proteins CCR5 and PD-1 are preserved on these hybrid exosomes, mediating active target homing toward melanoma cells [55]. Upon internalization, cytolytic molecules natively encapsulated within the γδ-T exosomes, including granzyme A, granzyme B, perforin, and granulysin, cooperatively induce specific tumor cell apoptosis without harming normal tissues [55]. When combined with 650 nm light irradiation, Ce6-mediated ROS generation synergizes with these cytolytic molecules, triggering massive melanoma cell death and a classic ICD response characterized by calreticulin exposure, HMGB1 leakage, and decreased intracellular ATP [55]. This biomimetic hybrid nanoplatform successfully promotes human DC maturation and drives potent MelanA/MART-1-specific CD4+ and CD8+ T-cell responses in humanized mice [55]. Finally, to resolve the intrinsic photophysical trade-off in conventional PS, where Type I radical generation and Type II singlet oxygen transfer competitively deplete the same triplet excited state, advanced metal-based biradical PS have been engineered [51]. The cyclomethylated Iridium(III) complex Ir-tBu2Tspi undergoes photo-induced intramolecular homolytic bond cleavage to generate a transient open-shell biradical intermediate [51]. This intermediate reconfigures the electronic structure of the molecular scaffold, combining a strongly reducing radical center with a quinoid-stabilized, long-lived triplet excited state [51]. Consequently, Ir-tBu2Tspi concurrently activates both Type I electron-transfer and Type II energy-transfer pathways, producing superoxide, hydroxyl radicals, and singlet oxygen with unprecedented quantum efficiency [51]. This extensive ROS flux triggers PANoptosis, an integrated, highly inflammatory programmed cell-death pathway that simultaneously engages apoptotic, necroptotic, and pyroptotic machinery via PANoptosome assembly [51]. When encapsulated in PEGylated lipid formulations (Ir-tBu2Tspi@PEG), intravenous administration followed by localized laser activation achieved a primary B16-F10 tumor inhibition rate of 90.6% and a distant, non-irradiated tumor inhibition rate of 95.3% [51]. This dual-pathway platform promotes massive splenic DC maturation (58.6%) and increases CD8+ cytotoxic T-cell infiltration into distant, untreated metastases to 21.5%, establishing biradical-mediated PANoptosis as a premier molecular design principle for systemic photodynamic immunotherapy [51].

### 8.3. Synergy with Immune Checkpoint Blockade (ICB) and Adoptive Cell Therapies (ACT)

Although localized PDT and Nano-PIT can successfully initiate the tumor-immune cycle by driving ICD and releasing TAAs, their systemic efficacy is frequently blunted by tumor-intrinsic resistance mechanisms and the induction of adaptive immune-suppressive checkpoints [12,54]. To overcome these hurdles, contemporary photoimmunotherapy focuses on combining local photodynamic destruction with systemic immunotherapies, specifically immune checkpoint blockade (ICB) and adoptive cell therapies (ACT) [12,52,54]. Combining local PDT with systemic ICB leverages a powerful therapeutic synergy: the localized photodynamic intervention acts as an autologous in situ vaccine that primes the immune system, while ICB “releases the brakes” on the newly generated cytotoxic T lymphocytes, preventing their systemic exhaustion in the TME [12,52,54]. For example, combining PDT with a pH-responsive polymeric nanobooster carrying Ce6 and anti-PD-L1 antibodies (NC@Ce6) promotes calreticulin exposure and DC maturation under 650 nm light [53]. In B16F10 melanoma-bearing mice, this combination significantly enhances tumor infiltration of CD3+ T lymphocytes and CD8+ CTLs (reaching 49%), yielding a tumor inhibition rate of 78% [53]. Similarly, the use of a dual-responsive polymeric micelle that hierarchically releases anti-PD-L1 and zinc phthalocyanine (ZnPc) at the acidic, metallopeptidase 2 (MMP-2)-rich tumor site ensures precise spatiotemporal delivery, maximizing local photodynamic ICD while minimizing systemic immune-related adverse events [12,54]. Other highly effective combination strategies utilize Near-Infrared Photoimmunotherapy (NIR-PIT), which conjugates the membrane-rupturing dye IRDye700DX (IR700) to tumor-targeting mAbs [12,52]. Anti-CD29-directed NIR-PIT selectively eradicates melanoma cells while completely sparing vital tumor-infiltrating immune populations, such as DCs, effector T cells, and NK cells [43]. When paired with systemic anti-CTLA-4 therapy, anti-CD29 NIR-PIT significantly prolongs mouse survival, upregulates CD8+ T-cell activation markers, and drives complete systemic clearance [12,52]. CD146-targeted NIR-PIT has similarly demonstrated strong localized singlet oxygen generation and tumor volume reduction [12,52]. Furthermore, bioengineered nanoplatforms can simultaneously address the biophysical and vascular barriers of the melanoma microenvironment to potentiate ICB [12,52]. The “Combo-NP” platform co-delivers a vascular endothelial growth factor receptor (VEGFR) inhibitor (lenvatinib) and pseudo-semiconducting polymers to simultaneously normalize tumor vasculature and generate ROS upon NIR activation [12,52]. This vascular normalization drastically improves systemic T-cell infiltration, alleviates hypoxia, and synergizes with systemic anti-PD-1 or anti-PD-L1 blockades to eradicate primary and metastatic lesions [12,52]. To bypass the ECM barrier that physically blocks immune cell entry, MN patches formulated with hyaluronidases (HAases) have been deployed to degrade localized HA, dramatically enhancing the penetration of Ce6-loaded Prussian blue nanoparticles and effector T cells, leading to complete primary tumor clearance and robust abscopal regression of distant tumors when combined with systemic anti-PD-1 [12,52]. Beyond ICB, PDT has emerged as a premier priming strategy for Adoptive Cell Therapies (ACT), which are frequently hindered in solid tumors by poor cellular trafficking and low infiltration [12,52,54]. Localized photothermal therapy utilizing PLGA nanoparticles loaded with ICG (PLGA-ICG) has been shown to dilate tumor microvasculature, inflame the TME, and stimulate the expression of chemokine profiles essential for T-cell homing [12,52]. Consequently, priming primary melanoma lesions with PLGA-ICG phototherapy prior to the infusion of chimeric antigen receptor T cells (CAR-T) targeting chondroitin sulfate proteoglycan-4 (CSPG4) results in a massive increase in CAR-T tumor trafficking, accumulation, and antigen-specific activation, successfully inhibiting solid xenograft recurrence [12,52,54]. Similarly, localized phototherapy using PEG-PLGA nanoparticles loaded with a polymeric NIR dye (NP-PBT) induces robust vascular permeability and NKT cell-recruiting chemokines. Priming melanoma-bearing mice with NP-PBT phototherapy prior to the adoptive transfer of ex vivo-expanded natural killer T (NKT) cells drives complete tumor clearance and establishes durable immunological memory [12,52].

### 8.4. Targeting Immunosuppressive Populations and TME Remodeling

While activating effector T lymphocytes is critical, a major barrier to successful melanoma photoimmunotherapy is the highly suppressive nature of the TME [12,54]. Solid melanoma lesions actively recruit immunosuppressive cell populations, specifically regulatory T cells (Tregs), myeloid-derived suppressor cells (MDSCs), and tumor-associated macrophages (TAMs), which systematically deactivate CTLs and drive resistance to standard immunotherapies [12,52,54]. Modern PIT has therefore designed active strategies to deplete these suppressive populations or metabolically reprogram the TME [12,54]. A highly selective strategy to reverse Treg-mediated immunosuppression utilizes targeted photodynamic depletion [12,52,54]. Since Tregs constitutively express high levels of the interleukin-2 receptor alpha chain (CD25), researchers developed an anti-CD25 monoclonal antibody conjugated directly to Chlorin e6 [12,52]. Intratumoral injection of this conjugate followed by localized light irradiation selectively destroys tumor-infiltrating CD25+ Tregs via localized singlet oxygen generation, without inducing systemic autoimmunity or depleting resting naïve T cells [12,52]. This precise Treg depletion leads to a massive, immediate influx of active CD8+ CTLs, increases IFN-γ secretion in the TME, and successfully reverses the checkpoint-mediated resistance that typically blunts systemic ICB outcomes [12,52]. Selective NIR depletion of polymorphonuclear MDSCs (PMN-MDSCs) has also been achieved using targeted NIR-PIT, significantly enhancing the host effector immune response [12,52]. Furthermore, PIT can be leveraged to block the adaptive biochemical defense pathways activated by tumors post-treatment [12,54]. Under photodynamic or photothermal stress, cancer cells and TAMs strongly upregulate the expression of indoleamine 2,3-dioxygenase (IDO), an intracellular rate-limiting enzyme that metabolizes essential tryptophan into kynurenine [12,54]. Local tryptophan depletion arrests CTLs in the G1 phase of the cell cycle and induces their apoptosis, while kynurenine accumulation differentiates naïve T cells into suppressive FoxP3+ Tregs, culminating in a highly tolerogenic TME [12,54]. To disrupt this negative feedback loop, Nano-PIT systems co-encapsulate a PS alongside an IDO inhibitor, such as NLG919 or 1-methyltryptophan [12,53,54]. For instance, PEG-PLGA nanoparticles co-loading NLG919 and the silicon-phthalocyanine ZnF16Pc accumulate in tumors and, upon light activation, trigger direct cell death while simultaneously blocking IDO activity, significantly increasing CD8+ CTL infiltration and animal survival [12,54]. In addition to cellular targeting, PIT has been engineered to counteract metabolic immunosuppression and inhibitory soluble DAMPs [12,13]. Stressed and dying cancer cells release high levels of cyclooxygenase-2 (COX-2) and prostaglandin E2 (PGE2), which function as “inhibitory DAMPs” that impair macrophage cytolytic cytokine production and promote tumor angiogenesis [12,13]. Prolonged blockade of the PGE2 pathway post-PDT using COX-2 inhibitors has been shown to significantly enhance PDT curative outcomes in pre-clinical models [12,13]. Similarly, the active enzymatic degradation of the ECM via hyaluronidases combined with pH-responsive nanocarriers allows the physical de-densification of the tumor stroma, converting a physically and immunologically “cold” tumor into an active, highly inflamed “hot” TME [12,54].

### 8.5. Clinical Evidence, Abscopal Effects, and Translational Benchmarks

The translation of photoimmunotherapy from bench to bedside remains a critical clinical goal [12]. The premier melanoma-specific clinical evidence retrieved by this search is represented by the pioneering work of Li et al., who conducted a phase I/II clinical trial evaluating the safety and therapeutic efficacy of in situ photoimmunotherapy (iSPi) in 11 late-stage metastatic melanoma patients [12,15]. The iSPi protocol consisted of three integrated components applied directly to cutaneous metastases: (1) twice-daily topical application of the FDA-approved TLR7 agonist imiquimod (5% cream) under plastic occlusion to prime the local stroma; (2) intratumoral injection of the NIR dye ICG at a dose of 0.5 mL/cm^3^; and (3) localized NIR 805 nm diode laser irradiation (1.0 W/cm^2^, 10 min) at the beginning of weeks 2 and 4 [12,15]. The clinical outcomes of this trial were highly encouraging: all patients successfully completed at least one 6-week cycle of treatment, and the regimen was exceptionally well-tolerated [15]. The most common treatment-related side effects were localized cutaneous reactions, specifically mild-to-moderate rash (90.9%), pruritus (81.8%), and pain (54.5%), with a complete absence of any Grade 4 systemic toxicities [15]. Out of the evaluable cohort, complete response (CR) was observed in 6 patients, with 8 patients achieving a complete local response (CLR) at the directly irradiated primary treatment sites [15]. Most importantly, complete local response was observed in non-treatment site (regional) lesions in 4 patients [15]. This regional regression of un-irradiated distant tumors provided the first direct clinical evidence of the abscopal effect in melanoma patients treated with photoimmunotherapy [12,15]. Mechanistically, the combination of imiquimod-mediated TLR7 signaling and laser-induced thermal tumor lysis promoted the massive recruitment and activation of Langerhans cells, macrophages, and NK cells, driving a systemic Th1-skewed adaptive cytotoxic response [12,15]. The 1-year overall survival probability for these advanced metastatic patients reached 70%, with five patients remaining alive and completely disease-free at the time of the last follow-up [15]. In parallel, preclinical nanomedicine has systematically validated these clinical mechanisms using advanced biomimetic virus-like platforms [12]. Nkanga et al. engineered a highly sophisticated, high-aspect-ratio nanotube platform utilizing the tobacco mosaic virus (TMV) [12,14]. The interior channel of TMV was covalently conjugated to the synthetic small-molecule TLR7 agonist 1V209 via copper-catalyzed click chemistry, and its exterior surface was coated with the photothermal biopolymer polydopamine (PDA), affording the multifunctional nanoconjugate 1V209-TMV-PDA [12,14]. In a highly aggressive, pigmented B16F10 dermal melanoma model in C57BL/6 mice, a single intratumoral injection of 1V209-TMV-PDA followed by 808 nm NIR laser irradiation (1 W/cm^2^, 5 min) achieved complete primary tumor ablation within 2 days [12,14]. Critically, while laser monotherapy resulted in rapid local recurrence and a median survival of only 25 days, 60% of the mice receiving the complete 1V209-TMV-PDA + laser combination remained alive and completely tumor-free at the 50-day study endpoint [12,14]. Systematic splenocyte analysis via ELISpot confirmed that this combined photothermal immunotherapy established a powerful systemic immunological memory, inducing a 2-fold increase in tumor-specific IFN-γ-secreting cytotoxic T lymphocytes when re-challenged with B16F10 cells [12,14].

## 9. Related Photo-Mediated Approaches: Chemo-Photothermal Systems and Photoacoustic Laser Killing

### 9.1. Delineating Boundaries Between Photochemical and Photothermal Modalities

In dermatologic oncology, light-based modalities must be strictly categorized by their primary energy conversion pathway to evaluate their therapeutic efficacy and translational potential accurately. Conventional photodynamic therapy (PDT) operates via a photochemical process wherein an excited photosensitizer transfers energy or electrons to molecular oxygen, generating cytotoxic ROS and singlet oxygen (O 12). Consequently, classical PDT is strictly dependent on the local partial pressure of oxygen (${\mathrm{pO}}_{2}$) and is inherently vulnerable to TME hypoxia. In contrast, PTT relies on photothermal transduction agents (PTAs) that convert absorbed photon energy directly into kinetic heat [56,57,58,59]. This non-photochemical mechanism operates independently of molecular oxygen, rendering PTT immune to hypoxia-induced resistance, but introduces distinct challenges, such as nonspecific thermal dissipation, risk of heat-shock protein (HSP) upregulation, and collateral thermal damage to surrounding healthy tissue. Furthermore, PIT leverages light-induced stress, either photochemical or photothermal, as a primary trigger to induce immunogenic cell death (ICD) and DAMP release, thereby converting localized injury into systemic adaptive immunity. Finally, photoacoustic (PA) therapies utilize ultrashort laser pulses to induce localized thermoelastic expansion and mechanical cavitation, exploiting endogenous chromophores (such as melanin) for direct physical cell lysis rather than chemical or thermal destruction. Recognizing these distinct biophysical foundations is essential, as the criteria for optimizing light dosimetry, nanocarrier delivery, and combination regimens vary fundamentally across these modalities [56,57,58,59].

### 9.2. Synergistic Chemo-Photothermal Systems (Chemo-PTT) in Melanoma

The integration of chemotherapy and photothermal therapy (chemo-PTT) within smart nanocarriers represents one of the most robust therapeutic paradigms in superficial skin cancers, achieving synergistic tumor eradication at reduced drug dosages [58,59,60,61]. In these systems, hyperthermia not only induces direct thermal damage but also alters tumor physiology by increasing local vascular perfusion, elevating tissue oxygenation, and destabilizing the polymer or lipid matrices of the carrier to trigger on-demand, accelerated drug release [61,62,63,64]. Furthermore, local heat increases cell membrane permeability, facilitating enhanced endocytosis of the chemotherapeutic payload and bypassing multidrug resistance (MDR) mechanisms [58,64].

#### 9.2.1. Lipopolymersomal and Liposome-Based Co-Delivery Platforms

The hybrid marriage of phospholipids and block copolymers within single vesicles provides a highly stable, biocompatible vehicle for co-encapsulating hydrophobic and hydrophilic agents with adjustable release kinetics [64,65]. Athena Abbasi et al. fabricated a nucleolin-targeted, multipurpose lipopolymersome consisting of poly(ethylene glycol)–poly(lactic acid) (PEG-PLA) and 1,2-dioleoyl-3-trimethylammonium propane (DOTAP) loaded with doxorubicin (DOX) and ICG [57,65]. To achieve selective homing, the surface was decorated with the AS1411 DNA aptamer, which specifically binds nucleolin overexpressed on melanoma cells [65]. Under 808 nm NIR laser irradiation, the encapsulated ICG undergoes efficient photothermal conversion, raising the local temperature by 12-15 °C to trigger cancer cell apoptosis [65]. This synergistic chemo-PTT platform (Apt-DOX-ICG-LP + Laser) achieved superlative tumor shrinkage and exceptional survival rates in B16F10 tumor-bearing mice compared to monotherapy or non-irradiated controls, while protecting healthy tissues from off-target toxicity [65]. Similarly, Hou et al. designed a core–shell thermo-nanoparticle (CSTNP) co-loaded with the dacarbazine-related alkylating agent temozolomide (TMZ) and the NIR ICG dye derivative IR820 (termed IT-CSTNPs) [64]. The CSTNPs comprised a dipalmitoyl phosphatidylcholine (DPPC) thermosensitive lipid shell and a biodegradable PLGA core, ensuring that local hyperthermia of 41.5–41.9 °C induced by the IR820 dye triggered a gel-to-liquid phase transition in the DPPC shell, resulting in an explosive, targeted release of TMZ inside A375 melanoma lysosomes [64]. Along the same line, Fazeli et al. developed an aptamer-targeted biomimetic construct in which gold nanostars coated with a mesoporous organosilica layer were loaded with doxorubicin, camouflaged with B16F0 cancer cell membranes and functionalized with the Sgc-8c aptamer; under 808 nm irradiation, this platform combined NIR-triggered drug release, chemo-photothermal cytotoxicity and computed tomography contrast enhancement, achieving complete tumor regression in melanoma-bearing mice [63].

#### 9.2.2. Two-Dimensional (2D) Nanosheets and Metal–Organic Frameworks

Two-dimensional inorganic nanostructures and NMOFs offer exceptionally high surface-to-volume ratios and uniform drug loading capacities [57,62]. Himanshu N. Bhatt et al. designed the “Bimodal Light Assisted Skin Tumor and Metastasis Treatment” (BLAST) platform, utilizing two-dimensional hexagonal boron nitride (BN) nanosheets functionalized with the BCL-2 inhibitor Navitoclax (NAVI) [62]. Under 980 nm NIR laser irradiation (1.2 W/cm^2^), the BLAST nanoparticles generate localized heat (up to 64–65 °C) that thermally degrades the ECM proteins, specifically collagen (COL1A1) and α-smooth muscle actin (α-SMA) [62]. This photothermal ECM remodeling reduces the tumor’s interstitial fluid pressure, paving a clear “tunnel” for NAVI to penetrate deep into the core of three-dimensional tumor spheroids and induce caspase-mediated apoptosis via BCL-2 downregulation [62]. Crucially, the light-triggered release of NAVI and the generation of ROS from the excited boron nitride lattice entered the systemic circulation, preventing and significantly reducing metastatic nodules in the lungs [62]. Taking a coordination chemistry approach, Mahsa Nazari et al. synthesized Fe-porphyrin-based Zr-metal–organic frameworks (FeP-Zr NPs) loaded with DOX and coated with an RGD-peptide-functionalized dextran shell (DOX@FeP-Zr/DEX/RGD) [57]. Under 808 nm laser irradiation, the Fe-porphyrin linkers act as highly stable photothermal transducers, elevating the temperature of B16F0 melanoma cells by 7 °C to induce localized thermal ablation [57]. The integration of Fe and Zr ions additionally endowed the platform with high contrast-generating capabilities for magnetic resonance imaging (MRI) and computed tomography (CT), enabling real-time, image-guided chemo-photothermal therapy that achieved 90% tumor eradication in vivo [57].

#### 9.2.3. Transdermal MN Patches with Stimuli-Responsive Nanoparticles

To bypass the stratum corneum and avoid the multi-organ toxicity of systemic administration, dissolvable MN patches have been integrated with stimuli-responsive nanoparticles for localized, pulsatile delivery [60,61]. Chenyuan Wang et al. developed a self-monitoring MN patch incorporating aggregation-induced emission (AIE)-active PATC microparticles co-encapsulating DOX and ICG (D/I@PATC) [60]. Upon 808 nm light irradiation (0.5 W/cm^2^), the encapsulated ICG generates localized heat, inducing a reversible phase transition and dissociation of the PATC microparticles, which triggers a pulsatile burst of DOX [60]. Due to the AIE and fluorescence resonance energy transfer (FRET) properties of the polymer, the drug release process can be directly monitored and verified in vivo via real-time fluorescence color changes at the tumor site, achieving a 97% tumor growth inhibition rate in preclinical in vivo melanoma mouse models [60]. In a similar study, Wanbing Qin et al. constructed a spatiotemporally controlled transdermal patch containing thermal-sensitive solid lipid nanoparticles (SLNs) loaded with paclitaxel (PTX) and the NIR dye IR-780 (PTX/IR-780 SLNs @DMNs) [61]. The SLN lipid matrix (tricaprin and cetyl palmitate) is engineered to undergo a solid-to-liquid phase transition at 50 °C, accelerating PTX release during laser “on” cycles, while rapidly re-solidifying when the laser is “off” to limit premature drug leakage [61]. This localized accumulation completely eradicated primary subcutaneous B16 tumors in vivo, yielding 100% local tumor regression in preclinical in vivo murine models over a 30-day observation period [61].

Other therapeutic MN combinations identified in this review include:

ZER-ICG-NMs@DMNs (Qing Zhu et al.): A hyaluronic-acid-functionalized dissolvable MN patch delivering polymeric micelles co-loaded with the natural sesquiterpenoid Zerumbone (ZER) and ICG [66]. Under NIR exposure, the synergistic system induces robust apoptotic cascades (upregulating Bax and Caspase-3) and significantly downregulates the expression of the melanoma marker Melan-A and pro-tumorigenic F4/80 M2 macrophages [66].

Cur NDs/IR820/HA MNs (Shan et al.): A two-layered hybrid MN composed of an embeddable, dissolving tip loading carrier-free curcumin nanodrugs and IR820, supported by a cross-linked sodium alginate/gelatin/hyaluronic acid (SA/Ge/HA) backing layer [67]. The MNs eradicate B16F10 melanoma through synergistic chemo-PTT, while the remaining backing layer covers the surgical defect, promoting the proliferation of endothelial cells and fibroblasts to accelerate skin regeneration [67].

CPT-CuS-ZIF-8@HA@MNs (Yiting Zhao et al.): A dissolving MN patch containing zeolitic imidazolate framework-8 (ZIF-8) nanoparticles co-loaded with camptothecin (CPT) and CuS photothermal nanoparticles [68]. The platform exploits CD44-receptor-mediated targeting and the pH/NIR dual-responsiveness of the HA-capped ZIF-8 shell, which dissociates in the acidic lysosomal microenvironment and undergoes photothermal collapse to trigger highly localized chemo-PTT [68].

#### 9.2.4. Alternative and Naturally Derived Nano-Systems

To enhance biocompatibility and avoid long-term toxicity, researchers have explored natural biopolymer-based carriers:

HA-PLGA/MXP NPs (Su Bin Lee et al.): Encapsulating 2D transition metal carbides (MXenes) and PTX within an amphiphilic matrix of HA and poly(lactide-co-glycolide) (HA-PLGA) [69]. MXenes’ exceptional light-to-heat conversion raises the local temperature above the glass transition temperature (Tg) of the PLGA polymer core, triggering sustained PTX release that achieves a 95.7% tumor inhibition rate in vivo [69].

PPyNPs-hydrogel (Hadis Veisi et al.): An injectable, thermosensitive hydrogel composed of cationic agarose and TMPO-oxidized lignocellulose, encapsulating deferasirox (DFX) iron-chelating nanoparticles loaded in polypyrrole (PPy) [58]. Under 1064 nm Nd:YAG laser irradiation, the hydrogel provides controlled, sustained DFX release through its microporous structure, achieving dose-dependent cytotoxicity against B16F10 melanoma cells [58].

DOX-ICG-cubo (Zhenzhen Chen et al.): Liquid crystalline glyceryl monooleate (GMO)-based cubosomes co-encapsulating DOX and ICG [59]. Under 808 nm laser irradiation, the cubosomes protect ICG from rapid photobleaching and degradation, while transdermal MN pre-treatment ensures deep skin penetration to achieve a remarkable 97.59% tumor inhibition rate in vivo [59].

MeNPs/DTIC (Motlagh-Haghnegahdar et al.): Natural melanin nanoparticles extracted from cuttlefish (Sepia officinalis) ink sacs loaded with dacarbazine (DTIC) through electrostatic interactions, offering an entirely biodegradable, non-toxic organic photothermal agent that promotes tumor regression and necrosis under 808 nm laser irradiation [70].

### 9.3. Photoacoustic Flow Cytography and On-the-Spot Laser Killing

Connecting the biology of metastatic dissemination with direct, physical light-mediated intervention, researchers have successfully converted melanin from a limiting barrier into an active, therapeutic target [19,71].

#### 9.3.1. In Vivo Label-Free Photoacoustic Flow Cytography and Selective Lysis of CTCs

Yun He et al. developed an innovative in vivo label-free photoacoustic (PA) flow cytography system integrated with a nanosecond-pulsed therapy laser to detect and destroy CTCs on the fly [19]. The technology exploits the intrinsic optical absorption contrast of melanosomes within melanoma CTCs compared to surrounding red blood cells (RBCs) [19]. While RBCs and CTCs absorb light similarly at 532 nm due to hemoglobin, excitation with a 1064 nm laser generates strong, selective PA signals from the pigmented melanosomes, producing a contrast-to-noise ratio of ~25 while RBC signals remain below the noise level [19]. Upon real-time hardware detection of a CTC-specific PA signal above a calibrated threshold, the system immediately triggers a coaxially aligned, nanosecond-pulsed 1064 nm therapy laser (25 J/cm^2^, 50 µm spot diameter) within ~10 µs [19]. This pinpoint laser pulse heats the melanosomes beyond the threshold for explosive vaporization, causing local photomechanical shockwaves that selectively lyse the CTC without causing collateral thermal damage to adjacent RBCs or blood vessels [19]. In pseudo-therapy metastatic models, this real-time CTC clearance achieved a high success rate, significantly reducing distant hematogenous tumor formation and metastasis [19].

#### 9.3.2. Ultrasound-Assisted Laser Therapy (USaLT)

To improve the depth and safety of light-mediated intervention without requiring any foreign therapeutic agents, Madhumithra Subramanian Karthikesh et al. pioneered Ultrasound-Assisted Laser Therapy (USaLT) [71]. USaLT combines low-fluence nanosecond pulsed lasers (532 nm or 1064 nm) with concurrently applied focused ultrasound (FUS) bursts (peak negative pressure of 2 MPa) [71]. Unlike transpupillary thermal therapy (TTT), photobiomodulation (PBM), or conventional PDT, which rely on continuous-wave lasers or chemical sensitizers [71], USaLT is primarily a mechanical, cavitation-driven approach [71]. The nanosecond laser pulse induces localized acoustic waves and transient cavitation bubbles in highly light-absorbing pigmented melanoma cells [71]. These bubbles are then forcefully driven by the applied ultrasound, triggering enhanced acoustic cavitation, shear stress, and microjetting that selectively destroy the melanoma membrane [71]. Ex vivo studies using a novel melanoma model established in chicken breast tissue demonstrated that USaLT selectively removed 66.41% of B16F10 melanoma cells at a low laser fluence (28 mJ/cm^2^ at 532 nm) [71]. Under 1064 nm irradiation, USaLT safely achieved effective melanoma destruction at a tissue depth of 3.5 mm, leaving adjacent non-pigmented muscle and connective tissue fibers completely intact (Table 2) [71].

**Table 2:** Comparative biophysical, photochemical, and clinical analysis of conventional PDT versus adjacent photo-mediated modalities in melanoma.

Modality	Primary Mechanism of Action	Oxygen Dependency (3O2)	Typical Sensitizers/Transducers	Key Advantage in Melanoma	Primary Translational Limitation
Conventional 1p-PDT	Type I/II photochemical generation of ROS and O 12	Strict dependency (O 32-dependent)	Porphyrins, Chlorins, Phthalocyanines (Pcs)	Non-invasive, spatially controlled, established in NMSC	Optical shielding by melanin, ROS scavenging, TME hypoxia
Femtosecond 2p-PDT	Multiphoton NIR absorption, FRET/radiative energy transfer	Moderate dependency	Visudyne (Verteporfin)	Converts melanin from an obstacle into an active energy mediator (>6-fold LD50 reduction)	Requires ultrafast femtosecond lasers, complex clinical hardware
Photothermal/Chemo-PTT	Light-to-heat transduction, thermal protein denaturation, drug release	Oxygen-independent	ICG, IR820, Gold/MXene/Boron Nitride nanosheets	Bypasses hypoxia, increases tissue perfusion, triggers localized pulsatile drug release	Risk of collateral thermal tissue damage, unstandardized thermal dosimetry
Photoimmunotherapy (Nano-PIT)	Immunogenic Cell Death (ICD), DAMP release, APC & T-cell priming	Variable (depends on PS/PTA carrier)	Ce6 + CpG, BP-PEG-R837, ICG + 1-MT, Ir(III) complexes	Converts local photic damage into systemic immunity, driving abscopal regression of metastases	Risk of immune evasion, TME immunosuppression, lack of RCTs
Photoacoustic Laser Killing	Selective photomechanical shockwaves and explosive melanosome vaporization	Oxygen-independent	Endogenous Melanin (label-free)	Real-time single CTC identification and lysis during blood transit without RBC damage	Technically complex, restricted to intravascular circulating tumor cells (CTCs)

### 9.4. Translational Outlook and Methodological Bridging

Despite the remarkable success of these advanced photothermal, chemo-photothermal, and photoacoustic platforms, a significant translational gap must be bridged before clinical deployment. Methodologically, the field is limited by a heavy reliance on static, two-dimensional (2D) monolayer cell models, which fail to replicate the microenvironmental complexity of solid tumors. Evaluating these complex nano-systems requires a systematic transition to three-dimensional (3D) multicellular tumor spheroids, which accurately mimic the in vivo cellular architecture, pH gradients, partial pressure of oxygen (pO_2_), and dense, collagenous ECM networks that act as major physical barriers to nanoparticle penetration [62]. Preclinical investigations have highlighted that while nanoparticles remain confined to the periphery of 3D spheroids under static conditions, the application of local light-to-heat conversion or USaLT-induced mechanical cavitation effectively disrupts the spheroid microenvironment, facilitating deep, core penetration of the therapeutic payload and providing highly relevant, predictive data for in vivo translation [62]. Furthermore, as the field transitions toward human trials, researchers must systematically distinguish conventional, oxygen-dependent PDT from these adjacent, thermally or photomechanically driven approaches. Standardizing dosimetry parameters, including laser wavelength, pulse width, fluence rate, tissue pigmentation, and thermal relaxation thresholds, will be critical to ensuring safety, mitigating phototoxic skin reactions, and successfully positioning these innovative biophysical strategies within the clinical oncology toolkit.

## 10. Safety and Translational Limitations

### 10.1. Safety Profiles, Acute Tolerability, and Dermatologic Side Effects

Before examining the specific safety profile of PDT in malignant melanoma, it is valuable to recognize that PDT has already established a robust track record of clinical safety, tissue preservation, and high patient tolerability across diverse, highly sensitive anatomical compartments [72,73,74]. In oral medicine and dentistry, PDT and photodynamic antimicrobial chemotherapy have proven effective and non-genotoxic for treating oral premalignant leukoplakia, mucosal infections, and head and neck squamous cell carcinomas, preserving healthy surrounding tissues with minimal scarring [72]. In ophthalmology, light-triggered regimens (such as Verteporfin- or Indocyanine Green-mediated therapies) are clinically validated for managing age-related macular degeneration, choroidal neovascularization, and intraocular tumors, achieving targeted vascular occlusion while protecting delicate retinal structures [73]. Similarly, in urological oncology, systematic evaluations of PDT for non-muscle-invasive bladder cancer confirm satisfactory therapeutic efficacy alongside a favorable safety profile, where adverse events are predominantly mild, transient (e.g., lower urinary tract symptoms), and easily manageable [74]. This broad, tissue-spanning safety foundation provides a solid rationale for expanding PDT into complex dermato-oncologic indications, provided that the distinct biophysical barriers of cutaneous melanoma, such as melanin optical shielding and core hypoxia, are systematically addressed [72,73,74]. While PDT is clinically established and internationally approved for treating superficial pre-cancerous and keratinocyte-derived malignancies, such as actinic keratoses, Bowen’s disease, and BCC, where it yields excellent cosmetic outcomes [2,5,75], defining its safety profile in malignant melanoma remains a complex task due to the limited availability of melanoma-specific clinical studies. In general, the localized generation of ROS and singlet oxygen during PDT triggers localized tissue damage, microvascular leakage, and acute inflammatory responses [75,76]. This inflammatory cascade manifests clinically as local erythema, edema, severe burning discomfort, and transient hyperpigmentation or crusting at the treatment site, which typically resolve within days or weeks [75,76]. Additionally, systemic or prolonged retention of PS in healthy tissues, particularly the skin, can cause cutaneous photosensitivity, necessitating strict protection from sunlight to avoid phototoxic reactions [4,75]. In the context of cutaneous malignant melanoma, local side effects can be more pronounced due to the aggressive nature of the tumor, which often requires higher PS concentrations, deeper tissue penetration, and increased light fluences compared to thin, superficial non-melanoma lesions [6,56]. Preclinical studies in hairless severe combined immunodeficient (SCID) mouse models have evaluated the acute tolerability of NIR laser-mediated PDT combined with targeted gold nanoparticles, reporting that while the combined regimen caused localized heat concentration and slight post-treatment erythema, it did not compromise overall animal welfare, induce abnormal behavioral changes, alter systemic organ weights, or cause detectable histological damage to vital internal organs like the liver, spleen, kidneys, heart, or lungs [56]. However, in clinical practice, achieving such high selectivity remains challenging, as off-target phototoxicity, structural damage to surrounding healthy dermal tissue, and severe local inflammation remain significant safety concerns that must be meticulously managed [3,4,6].

### 10.2. Pain Management and Photophysical Dosimetry

Pain is widely recognized as the most significant patient-limiting adverse effect during conventional PDT, particularly under red light illumination [2,75]. This acute pain is primarily driven by the rapid, massive generation of singlet oxygen, which induces immediate oxidative stress, lipid peroxidation of plasma membranes, and direct stimulation of local dermal nerve endings [75,76,77]. In conventional dermatologic PDT for actinic keratoses and thin skin carcinomas, several strategies have been deployed to mitigate this discomfort, including the use of cold air analgesia, local nerve blocks, or transitioning to Daylight PDT (DL-PDT) [2,5]. Daylight PDT leverages continuous, low-intensity natural daylight to steadily activate PpIX as it is synthesized, preventing the explosive accumulation of ROS and making the treatment virtually painless [2,75,77]. However, natural daylight or indoor daylight devices lack the spectral energy and penetration depth necessary to reach deep-seated melanomas [5,6]. To optimize therapeutic outcomes in thicker or deeper tumors while maintaining an acceptable safety profile, researchers have turned to advanced photophysical dosimetry, such as light fractionation and pulsed laser systems [75,76]. Light fractionation involves dividing the total light dose into two or more discrete exposures separated by a specific dark interval [76]. Preclinical and clinical studies, particularly using 5-ALA, have demonstrated that fractionated schemes significantly improve tumor control and lesion clearance rates compared to continuous single-dose illumination [76]. This superior efficacy is biochemically driven by the kinetics of tissue oxygenation and PS dynamics [76]. Continuous high-fluence illumination rapidly depletes localized molecular oxygen, leading to temporary tumor hypoxia that halts Type II singlet oxygen generation [76]. Introducing a dark interval (typically 1 to 2 h) allows for microvascular reoxygenation of the tumor tissue and continued enzymatic synthesis of PpIX, leading to a second, highly destructive wave of ROS production upon subsequent light exposure [76]. Despite these advantages, establishing a standardized clinical protocol for fractionated PDT remains difficult due to the wide heterogeneity in PS formulations, cumulative light doses, and dark interval durations across the literature [76]. To address the optical heterogeneity of light sources reported across melanoma photomedicine, a systematic categorization of laser wavelengths, tissue penetration capabilities, and associated photosensitizers or transducers is essential. Table 3 provides a consolidated photophysical map detailing the spectrum from short-wavelength visible light to the second near-infrared (NIR-II) windows and ultrafast multiphoton activation, outlining their primary mechanisms, melanin interactions, and therapeutic applications.

### 10.3. Biophysical and Biochemical Translational Barriers

The clinical translation of conventional PDT for melanoma is fundamentally obstructed by two major biophysical and biochemical barriers: melanin interference and tumor hypoxia [4].

Melanin Shielding and ROS Scavenging: Melanin pigment is a highly efficient natural chromophore that exhibits a broad, continuous absorption spectrum peaking in the ultraviolet range but extending strongly across the visible spectrum up to 700 nm [5,6]. This optical profile directly competes with PS for photon capture, severely restricting light penetration and preventing sufficient excitation of PS located in deeper tumor layers [5,6]. Beyond this biophysical shielding, melanin acts as a potent endogenous biochemical antioxidant and free-radical scavenger, rapidly neutralizing singlet oxygen and other reactive species before they can initiate lethal intracellular damage [6]. Furthermore, melanosomes can physically sequester PS within inactive compartments, preventing their localization to critical organelle targets like the mitochondria or ER, which reduces treatment-induced apoptosis [3,6]. To bypass these barriers, researchers have utilized NIR-activated PS (which operate above 700 nm where melanin absorption is lower) [5,6], depigmentation strategies using melanin synthesis inhibitors like phenylthiourea (PTU) [3,6], or advanced femtosecond pulsed lasers that can photophysically activate melanin itself as a therapeutic target [75].

Tumor Hypoxia: The core mechanism of conventional Type II PDT is strictly oxygen-dependent [75,76]. However, solid melanoma tumors are characterized by rapid cell proliferation, disorganized vasculature, and a highly hypoxic microenvironment [4,6]. As a result, the scarce molecular oxygen in the tumor core is quickly consumed during the initial phase of light irradiation, leading to treatment resistance, incomplete tumor destruction, and a high risk of local recurrence [4,76,77]. To address this “Achilles’ heel” of PDT, advanced nanomedicine strategies have introduced oxygen-carrying nanovectors (such as perfluorocarbons) [6,75], oxygen-independent PS (such as Type I-initiating bacteriochlorin-based metal–organic frameworks) [77], or cascade platforms that combine PDT with hypoxia-activated prodrugs like tirapazamine (TPZ), which selectively eradicate hypoxic tumor cells surviving the photodynamic treatment [4,75].

### 10.4. Nanomedicine Safety and Delivery Challenges

To overcome the inherent hydrophobicity, poor water solubility, and rapid self-aggregation of conventional second-generation PS, researchers have engineered a diverse array of nanocarriers, including liposomes, polymeric nanoparticles, dendrimers, and inorganic frameworks [3,6,78]. While these nanovectors improve pharmacokinetics and facilitate passive tumor accumulation via the EPR effect, they introduce new translational safety and regulatory challenges [3,6]. Organic systems (such as PLGA or lipid-based nanoparticles) are generally highly biocompatible and biodegradable, but they can still exhibit variable drug release profiles and physical instability in physiological fluids [3,6]. On the other hand, inorganic platforms (such as gold, upconversion, or silica nanoparticles) offer outstanding stability and easy surface modification for active targeting, but they raise significant concerns regarding long-term biosafety, tissue accumulation, and hepatic or renal clearance [3,6]. Furthermore, the physical dimensions and surface charge of these nanocarriers heavily dictate their biological behavior and potential toxicities [3,6]. For instance, preclinical studies have noted that larger nanoparticles (exceeding 150 nm) can exert a physical “asphyxia” effect on both cancer and healthy cells, causing non-specific cell death and a reduction in cell viability even under dark conditions [56]. Porphyrin-based nanoparticles and related formulations can also display unexpected dark toxicity and redox vulnerability at high concentrations, highlighting the critical importance of precise dose optimization [78]. Actively targeted nanoplatforms, which conjugate PS to mAbs or peptide ligands to selectively bind melanoma biomarkers (such as CD44, MIA, or integrins), offer the highest degree of specificity [3,6,56]. However, the clinical translation of these targeted “smart drugs” is severely hampered by their high manufacturing costs, the complexity of large-scale production, and the rigorous characterization required to ensure that the targeting ligands do not undergo structural degradation during processing, which could trigger severe off-target toxicities and immunogenic reactions [3,6].

### 10.5. Methodological Limitations and the 2D vs. 3D Translational Gap

A primary methodological limitation of the current melanoma-relevant literature is its overwhelming reliance on conventional two-dimensional (2D) in vitro monolayer cell cultures [6]. In 2D cultures, cancer cells are grown as static, single-cell layers directly exposed to a uniform environment with a steady, unrestricted supply of oxygen and nutrients [6]. Under these artificial conditions, PS and nanoparticles can easily penetrate cell membranes without encountering any physical barriers, leading to highly inflated and unrealistic therapeutic responses [6]. In solid human tumors, however, malignant melanocytes grow and proliferate within a complex three-dimensional (3D) tissue architecture, surrounded by the ECM, supportive stromal cells, and abnormal blood vessels [6]. Nanoparticles administered in vivo face severe biophysical obstruction, as they must navigate the dense collagen networks of the ECM and high interstitial fluid pressure to reach the tumor cells [6]. To bridge this substantial translational gap, researchers have developed multicellular 3D tumor spheroids that closely recapitulate the microenvironmental barriers, nutrient gradients, physical penetration resistance, and hypoxic regions characteristic of solid tumors in vivo [6]. Evaluating PDT in these advanced 3D platforms has revealed a significant drop in therapeutic efficacy compared to 2D models, highlighting the critical necessity of testing novel targeted nanocarriers in 3D settings before initiating animal studies [6]. While animal models (such as mouse xenografts) remain indispensable for evaluating systemic pharmacokinetics, safety, and therapeutic responses, they are extremely expensive, time-consuming, and carry inherent immunological and genetic differences (as they contain non-human host cells) that prevent them from fully mimicking human disease pathology. Therefore, the systematic deployment of standardized, human-derived 3D tumor spheroids is essential to expedite preclinical screening, optimize dosimetry, and maximize the success rate of future clinical trials [6].

### 10.6. The Clinical Translation Block and the Absence of RCTs

To critically evaluate the translational maturity of melanoma photomedicine, available scientific literature must be explicitly stratified across experimental tiers, rigorously weighing the relative strength, methodological quality, and clinical applicability of the evidence:

1. Preclinical In Vitro Evidence (High Mechanistic Strength, Low Translational Predictability): The vast majority of published literature rests on 2D monolayer cell cultures (e.g., A375, B16F10). While these models provide robust, highly reproducible mechanistic insights into photosensitizer quantum yields, reactive oxygen species (ROS) cascades, and organelle-specific photodamage, their translational quality is intrinsically weak. Two-dimensional cell layers completely lack the complex biophysical barriers of solid tumors, such as dense ECM networks, interstitial fluid pressure, and melanin optical shielding. Emerging studies utilizing 3D multicellular tumor spheroids offer higher methodological validity by reproducing localized oxygen, pH, and drug penetration gradients, though their standardized implementation across photomedicine remains limited.
2. Preclinical In Vivo Evidence (Moderate Strength, High Conceptual Proof-of-Concept): Animal models (primarily subcutaneous or metastatic murine models in C57BL/6 or immunocompromised mice) provide strong proof-of-concept evidence for advanced delivery systems, including targeted nanocarriers, transdermal microneedle arrays, multiphoton excitation, and photoimmunotherapy. These investigations convincingly demonstrate local tumor growth inhibition, microenvironmental hypoxia relief, and systemic CD8+ T-cell-mediated abscopal regression. However, the quality and clinical extrapolability of this evidence are constrained by major anatomical and physiological species differences: rodent skin possesses a significantly thinner epidermal layer, much higher hair follicle density, and distinct viscoelastic properties compared to human skin, which artificially alters microneedle insertion kinetics, light penetration, and nanocarrier retention.
3. Clinical Human Evidence (Low Level of Evidence, High Translational Gap): Human clinical evidence remains sparse, highly heterogeneous, and methodologically restricted to low-sample-size single-arm pilot studies, Phase I/II trials, and individual case series. Notable clinical benchmarks include in situ photoimmunotherapy (iSPi) in late-stage cutaneous metastases (Phase I/II) and Verteporfin-mediated PDT in posterior pole choroidal melanoma. While these studies demonstrate initial safety, partial-to-complete local tumor response, and preliminary systemic immune activation, they represent Level IV/V clinical evidence. Crucially, the field suffers from a complete absence of prospective, multi-center randomized controlled clinical trials (RCTs; Level I/II evidence) directly comparing light-based interventions against current gold standards, such as wide surgical excision, BRAF/MEK targeted therapies, or systemic immune checkpoint blockade. Consequently, while preclinical proof-of-concept evidence is extensive and mechanistically compelling, the strength and quality of clinical evidence remain insufficient to support standard bedside implementation, underscoring an urgent imperative for biomarker-guided, prospective clinical trials. A consolidated overview of these core unresolved bottlenecks, along with their key underlying mechanisms and required translational solutions, is provided in Table 4.

## 11. Conclusions

In conclusion, the advancement of melanoma photomedicine rests on three core pillars: First, conventional oxygen-dependent PDT remains clinically immature for malignant melanoma due to formidable biophysical barriers, including optical shielding and free-radical scavenging by endogenous melanin, restricted light penetration, and TME hypoxia. Second, bioengineered nanodelivery systems and advanced photo-mediated modalities offer unprecedented potential to overcome these limitations. Third-generation targeted nanocarriers and transdermal dissolving microneedles effectively enhance drug penetration and bypass the stratum corneum, while femtosecond two-photon excitation converts melanin into an active energy mediator, hypoxia-defying platforms sustain therapeutic activity in oxygen-depleted cores, and PIT triggers ICD to elicit systemic antitumor responses against distant metastases. Third, a substantial translational gap persists between preclinical proof-of-concept findings and clinical implementation. Translating these technological breakthroughs into routine dermato-oncology will require shifting preclinical validation to standardized 3D tumor spheroids, establishing precise photophysical dosimetry, identifying predictive response biomarkers, and conducting prospective randomized controlled trials to establish definitive clinical efficacy.

## 12. Future Directions

To successfully advance photodynamic photomedicine from bench to bedside, future research must execute a coordinated roadmap that bridges fundamental preclinical evaluation, engineering scale-up, and clinical trial design. Methodologically, preclinical validation must systematically transition from static 2D cell monolayers toward standardized 3D multicellular tumor spheroids, organ-on-a-chip devices, and humanized patient-derived xenograft models. These advanced tissue architectures are essential to accurately reproduce the high interstitial fluid pressure, dense collagenous stroma, and localized oxygen gradients characteristic of solid melanoma in vivo. Concurrently, rigorous standardization of photophysical dosimetry remains imperative. Establishing clear clinical guidelines across diverse light sources, fluence rates, pulse durations, and fractionated delivery schemes, alongside optimizing ultrafast femtosecond pulsed lasers, will be critical to maximize singlet oxygen quantum yields while minimizing procedural pain and off-target phototoxicity. From a diagnostic and translational perspective, integrating spatial transcriptomics with real-time liquid biopsy metrics, such as circulating tumor cell dynamics and exosomal microRNA signatures, will be essential to construct validated algorithms for biomarker-guided patient stratification. Defining these molecular criteria will enable clinicians to identify specific patient subsets most likely to benefit from cPDT action, smart nanocarrier delivery, or synergistic photoimmunotherapy. In parallel, industrial and engineering efforts must overcome key scale-up bottlenecks by establishing sterile, reproducible, and cost-effective manufacturing protocols for multi-layered transdermal microneedle arrays and smart nanoplatforms, backed by thorough long-term biosafety, tissue accumulation, and clearance profiling. Ultimately, the successful integration of phototherapy into standard oncologic practice hinges on initiating large-scale, multi-center prospective randomized controlled trials. Directly comparing targeted light-based strategies against current gold standards, such as wide surgical excision, targeted BRAF/MEK inhibitors, and systemic immune checkpoint blockade, will provide the definitive clinical evidence required to demonstrate local control rates and overall survival benefits in patients with malignant melanoma.

## Figures and Tables

**Table 3:** Systematic overview of light wavelength bands, tissue penetration depths, photosensitizers/transducers, and therapeutic applications in melanoma photomedicine.

Wavelength Spectrum/Band	Estimated Penetration Depth	Primary Photosensitizers (PS)/Transducers (PTA)	Melanin Interaction & Optical Behavior	Primary Mechanisms & Clinical/Preclinical Applications
UV-A/Violet (335–425 nm)	<0.5 mm (Epidermal)	Endogenous PpIX (Soret band), Curcumin	Maximum melanin absorption and optical shielding; heavy ROS scavenging.	Limited to superficial epidermoid lesions; high phototoxic potential.
Blue Light (450–480 nm)	0.5–1.0 mm (Shallow Dermal)	3MeTARF, Cu@Ferrihydrite (M-Cu@Fh)	High melanin shielding; exploited for photoinduced charge transfer.	Blue-light photoredox, MAPK activation, and dual ferroptosis/cuproptosis induction.
Green Light (520–540 nm)	1.0–2.0 mm (Upper Dermis)	Eosin, Eosin-DOTAGA, SCC-NPs	Moderate-to-high melanin absorption; used in Cherenkov CRET platforms.	Cherenkov Radiation-induced PDT (CR-PDT) utilizing internal radionuclides (^18^F, ^68^Ga) to bypass external light limits.
Red Light (630–670 nm)	2.0–6.0 mm (Deep Dermis)	Second-generation Chlorins (Ce6, Ppa), Pcs, ZnBP(w)	Moderate melanin competition; standard photodynamic therapeutic window.	Conventional Type II singlet oxygen (^1^O_2_) PDT, dissolving microneedle delivery, and hypoxia-activated cascade regimens.
NIR-I Band (750–900 nm)	6.0–10.0 mm (Subcutaneous)	ICG, IR780, IR820, Gold nanostars, BP nanosheets	Low melanin absorption; high tissue transparency.	Deep-tissue PTT, chemo-PTT, transdermal microneedle photothermal lysis, and photoimmunotherapy (PIT).
NIR-II Band (>1000 nm)	>10.0 mm (Deep Tissue)	Polypyrrole (PPy) hydrogels, Organic heterojunctions (PP NPs)	Minimal melanin extinction; negligible tissue scattering.	Ultra-deep PTT ablation and label-free photoacoustic circulating tumor cell (CTC) laser lysis.
Femtosecond Pulsed NIR (~800 nm, 100 fs)	Tunable/Nonlinear Focus	Visudyne (Verteporfin)	Ultrafast 2P absorption by melanin; converts melanin into an active FRET energy donor.	Femtosecond 2P-PDT achieving >6-fold cytotoxicity in pigmented melanotic melanoma vs. amelanotic lines.

**Table 4:** Consolidated summary of core unresolved biophysical, biological, pharmacotechnical, and clinical bottlenecks in melanoma photomedicine.

Translational Bottleneck	Primary Mechanism/Driver	Main Clinical & Biological Impact	Required Translational Solutions
Melanin Shielding & ROS Scavenging	Broad optical absorption (400–700 nm) and antioxidant radical stabilization by endogenous melanin.	Limits photosensitizer photon absorption in deep layers; neutralizes generated ROS.	Multiphoton 2P-PDT, NIR-II light activation (>1000 nm), depigmentation pretreatments.
TME Core Hypoxia	Severe oxygen scarcity (${\mathrm{pO}}_{2}<10\;\mathrm{mmHg}$) and rapid light-driven O 32 depletion.	Halts oxygen-dependent Type II singlet oxygen (O 12) generation.	Oxygen-generating nanovectors, hypoxia-activated prodrug cascades (e.g., TPZ), Type I/photoredox systems.
Light Penetration & Dosimetry	High photon scattering in dermal collagen; non-uniform light distribution.	Incomplete deep-tissue tumor destruction; risk of procedural pain and thermal necrosis.	Standardized fractionated delivery, light-emitting transdermal patches, real-time optical dosimetry.
Nanocarrier Biodistribution & Toxicity	Off-target accumulation; slow clearance of inorganic cores; variable dark toxicity.	Long-term organ retention (liver/spleen); potential systemic or dark phototoxicity.	Fully biodegradable organic/biomimetic vectors, comprehensive long-term ADME profiling.
Manufacturing & Scalability	High structural complexity of multi-layered microneedles and targeted nanoconjugates.	Batch-to-batch variability; high production costs; batch sterility challenges under GMP.	Scalable microfluidic assembly, standardized quality-by-design (QbD) formulation protocols.
Patient Selection & Biomarkers	Absence of validated prognostic or predictive biomarkers for phototherapeutic response.	Inability to stratify patients who will benefit from PDT vs. PTT/PIT vs. standard regimens.	Integration of spatial transcriptomics, liquid biopsies (CTCs/ctDNA), and multiplex tissue profiling.

## Data Availability

No new data were created or analyzed in this study. Data sharing is not applicable to this article.

## Abbreviations

The following abbreviations are used in this manuscript:

PDT	Photodynamic Therapy
NMSCs	Non-melanoma skin cancers
BCC	Basal cell carcinoma
cSCC	Cutaneous squamous cell carcinoma
ROS	Reactive oxygen species
ECM	Extracellular matrix
5-ALA	5-aminolevulinic acid
2p-PDT	Multiphoton photodynamic therapy
NIR	Near-infrared
FRET	Förster resonance energy transfer
CTPs	Circulating tumor products
CTCs	Circulating tumor cells
cfDNA	Cell-free DNA
ctDNA	Circulating tumor DNA
GEP	Gene expression profiling
IF-guided LCM	Immunofluorescence-guided laser capture microdissection
PS	Photosensitizer
HpD	Hematoporphyrin derivative
Pcs	Phthalocyanines
CR-PDT	Cherenkov Radiation induced PDT
CRET	Cherenkov Radiation Energy Transfer
3MeTARF	3-methyltetraacetyl-riboflavin
PLGA	Poly(lactic-co-glycolic acid)
EPR	Enhanced permeability and retention
PpIX	Protoporphyrin IX
ICG	Indocyanine green
NMOFs	Nanoscale metal–organic frameworks
CQDs	Carbon quantum dots
Fe-CDs	Fe ions-doped carbon dots
GSH	Glutathione
CH-SCC NPs	Sodium copper chlorophyllin-loaded chitosan nanoparticles
mAbs	Monoclonal antibodies
CSPG4	Chondroitin Sulfate Proteoglycan 4
MIA	Melanoma Inhibitory Activity
HA	Hyaluronic acid
scFvs	Single-chain variable fragments
AgNPs	Silver nanoparticles
SNA	Sambucus nigra lectin
MN	Microneedle
ICD	Immunogenic cell death
DAMPs	Danger-associated molecular patterns
TME	Tumor microenvironment
ER	Endoplasmic reticulum
DCs	Dendritic cells
TLR4	Toll-like receptor 4
ICAM-1	Intercellular adhesion molecule 1
TAAs	Tumor-associated antigens
MHC	Major histocompatibility complex
